# Constipation in Tg2576 mice model for Alzheimer’s disease associated with dysregulation of mechanism involving the mAChR signaling pathway and ER stress response

**DOI:** 10.1371/journal.pone.0215205

**Published:** 2019-04-12

**Authors:** Ji Eun Kim, Jin Ju Park, Mi Rim Lee, Jun Young Choi, Bo Ram Song, Ji Won Park, Mi Ju Kang, Hong Joo Son, Jin Tae Hong, Dae Youn Hwang

**Affiliations:** 1 Department of Biomaterials Science, College of Natural Resources & Life Science/Life and Industry Convergence Research Institute, Pusan National University, Miryang, Korea; 2 Department of Life Science and Environmental Biochemistry, College of Natural Resources & Life Science/Life and Industry Convergence Research Institute, Pusan National University, Miryang, Korea; 3 College of Pharmacy, Chungbuk National University, Chungju, Korea; Chinese Academy of Sciences, CHINA

## Abstract

**Background:**

Although constipation has been researched in various neurological disorders, including Parkinson’s disease (PD) and spinal cord injury (SCI), the pathological mechanism of this symptom has not been investigated in Alzheimer’s disease (AD) associated with loss of nerve cells in the brain. This study was undertaken to gain scientific evidences for a molecular correlation between constipation and AD.

**Methods:**

To understand the etiology, we measured alterations in various constipation parameters, muscarinic acetylcholine receptors (mAChRs) and endoplasmic reticulum (ER) stress response, in 11-month-old Tg2576 transgenic (Tg) mice showing AD-like phenotypes.

**Results:**

A high accumulation of amyloid beta (Aβ) peptides, a key marker of AD pathology, were detected in the cortex and hippocampus of Tg mice. Furthermore, significant alterations were observed in various constipation parameters including stool weight, histological structure, cytological structure and mucin secretion in Tg2576 mice. Moreover, M2 and M3 expression and the downstream signaling pathways of mAChRs were decreased in the Tg group, as compared with non-Tg (NT) group. Furthermore, activation of ER stress proteins and alteration of ER structure were also detected in the same group.

**Conclusions:**

The results of the present study provide strong novel evidence that the neuropathological constipation detected in Tg2576 mice is linked to dysregulation of the mAChR signaling pathways and ER stress response.

## Introduction

Constipation is an acute or chronic gastrointestinal disease characterized by infrequent bowel movements, hard and dry feces, incomplete bowel evacuation and difficulty during defecation [1–3]. Clinically, this disease is diagnosed with physical examinations (including feces for analysis) and careful rectal examination, as well as four key physiological examinations such as colonic-transit testing, anorectal manometry, balloon expulsion and defecography [4, 5]. Also, various physiological, genetic and environmental factors may contribute towards acute and chronic constipation. In most cases, acute constipation is induced by bowel obstruction, adynamic ileus and some drug administrations, whereas chronic constipation could result due to colonic tumors, metabolic disorders, central nervous system disorders, peripheral nervous system disorders, systemic disorders, functional disorders, and dietary factors [6–7].

Many neurological disorders are associated with symptoms of constipation. Studies on the location of lesions, the possible pathogenesis of symptoms, and therapeutic treatment, are rapidly expanding to better understand the physiology of bowel movements and defecation [8]. Among the several disorders affecting the brain, PD is a frequent and occasionally dominant disease accompanying gastrointestinal dysfunction including drooling, dysphagia, gastroparesis and constipation [9, 10]. More than 50% of PD patients suffer from moderate to severe constipation [11, 12]. However, no studies have demonstrated either a clinical or pathological association of constipation in stroke. Furthermore, although constipation and fecal incontinence coexist in 39–73% of multiple sclerosis (MS) patients, their pathological mechanism is poorly investigated [13, 14]. Bowel dysfunctions, such as altering diarrhea and constipation, are observed as a sign of peripheral nerve damage in generalized and pure autonomic neuropathies resulting from diabetes, amyloidosis and autoimmune disorders [15, 16]. However, there are no studies that associate constipation with AD, a very common chronic neurodegenerative disease, although constipation has been observed in 4.3–17.2% of AD patients [17, 18].

Due to their implications in numerous physiological and pathological functions of the brain, mAChRs are considered as suitable drug targets for the treatment of AD [19]. Activation of mAChR M1 by agonists induces various pharmacological effects that include reduced Aβ production [20], promoting α-secretase activity [21], decreasing BACE1 [22], inhibition of Aβ-induced neurotoxicity [23], and reduced tau phosphorylation [24]. One study revealed that the drug N-desmethylclozapine (NDMC), which possesses good pharmacological alleviation activity, exerts its M2 mAChR agonistic effect only in rats, whereas the M4 mAChR antagonism was observed only in humans [25]. These previous studies provide some clues about the correlation between AD and mAChRs.

Furthermore, there are conflicting reports regarding the ER stress response in various AD animal models, although the enhancement of ER stress response has been detected in post-mortem human AD brains [26, 27]. Upregulation of ER stress markers such as GRP78, CHOP, p-eIF2α and PDI were observed in APP/PS1, rTg4510, rTg21221, APP/PSEN1/Mapt and TauPS2APP models at various ages [28–32]. However, their levels were consistently maintained in other models including AppNL-G-F, Tg2576, APP23 and P3015-Tg models [28, 29, 33]. These results do not clearly indicate the involvement of ER stress in the pathogenesis of AD. Therefore, further research is necessary to verify the differences observed in various tissues.

The present study was therefore undertaken to identify scientific evidence for the pathological mechanism of constipation in AD, through analysis of various constipation parameters, protein activation of the mAChR signaling pathway, and alteration of ER stress response markers in an AD model showing amyloid plaque and neuronal cell loss. Our results primarily suggest the possibility that bowel movement and defecation in the pathogenesis of AD are associated with dysregulation of the mAChR signaling pathway and ER stress response.

## Materials and methods

### Care and use of animals and collection of tissue

Tg2576 mice were generated at the University of Minnesota, by microinjecting the APP695sw gene into B6SJLF2 zygotes. These mice showed a high concentration of Aβ peptides, amyloid plaques and memory deficits at age 9–10 months (S1A Fig)[34]. Adult Tg mice and B6SJL (Non-Tg, NT) mice used in this study were purchased from Samtaco BioKorea Co. (Osan, Korea), Taconic branch. The animal protocol for this study was reviewed and approved by the Pusan National University-Institutional Animal Care and Use Committee (PNU-IACUC; Approval Number PNU-2011-00220). All mice were handled at the Pusan National University Laboratory Animal Resources Center, which is accredited by the Korea Food and Drug Administration (FDA) (Accredited Unit Number-000231) and AAALAC International (Accredited Unit Number; 001525). The mice were provided with a standard irradiated chow diet (Purina Mills Inc., Seoungnam, Korea) *ad libitum*, and were maintained in a specific pathogen-free state under a strict light cycle (lights on at 08:00 h and off at 20:00 h) at 23±2°C and relative humidity 50±10%.

Eleven-month-old Tg2576 mice (n = 20: male = 10, female = 10) and NT mice (n = 20: male = 10, female = 10) were assigned to the Tg group or NT group, respectively. After fasting for 24 hr, all animals were immediately sacrificed using CO_2_ gas, following which blood was collected, and tissue samples were harvested and stored in 10% formalin solution; all samples were stored in Eppendorf tubes at -70°C, until assayed.

### Genotyping of Tg mice

To differentiate the Tg mice from NT mice, genomic DNA isolated from mouse tails were genotyped by polymerase chain reaction (PCR) analysis. For DNA-PCR, 10 pmol each of APP_SWE_-specific primers, sense: 5’-CTG ACC ACT CGA CCA GGT TCT GGG T-3’ and antisense: 5’-GTG GAT AAC CCC TCC CCC AGC CTA GAC CA-3’, were added into the genomic DNA template mixtures. The reaction mixtures were subjected to 25 cycles of amplification conducted in a T100 thermal cycler (BioRad Laboratories Inc., Hercules, California, USA) under the following conditions: denaturation for 30 sec at 94°C, annealing for 30 sec at 62°C, and extension for 45 sec at 72°C. The amplified PCR products were then loaded onto 1% agarose gel, after which the bands were detected using the Kodak Electrophoresis Documentation and Analysis System 120 (Eastman Kodak, Rochester, NY, USA)(S1B Fig).

### Morris water maze test

The Morris water maze experiment was conducted in a circular pool (diameter 180 cm, height 75 cm) with water maintained at 21±2°C. The pool was divided into quadrants and the hidden escape platform was placed 1 cm below the water surface in one of the quadrants. During the next 5 days, the mice swam three times a day (180 seconds each) over the platform. In the event of discovering the platform, the mice were allowed to remain on the platform for 60 seconds. The time required to find the hidden platform (latency) during each test period was recorded using a video tracking system. After the 5 day training session, the swimming time of the mouse was recorded on the 6th day with the escape platform removed from the pool.

### Analysis of food intake, water intake and body weight

Alterations in food intake, water consumption and body weight of Tg mice and NT mice were measured daily at 10:00 am throughout the experimental period, using a measuring cylinder and an electrical balance, respectively. All measurements were performed three times to ensure accuracy.

### Measurement of excretion parameters

To collect pure stools and urine without any contamination, all mice were bred in metabolic cages (Daejong Instrument Industry Co., LTD, Seoul, Korea) during the experimental period. The stools excreted from each Tg and NT mice were collected every day at 10:00 am. Stools were weighed three times per sample using an electric balance, whereas the water content was defined as the weight of water in stools, calculated by subtracting dry weight from wet weight of stools [35, 36]. The urine volume was measured three times per sample using a cylinder.

### Immunohistochemical analysis for brain

Perfusion test and Nissl staining of the brain were performed as previously described [37]. Briefly, NT and Tg mice were anesthetized by intraperitoneal injection of Zoletile (50 mg/kg body weight), after which they were transcardially perfused with 1× PBS followed by 4% formaldehyde, to effectively remove blood and fix the brain tissue. Following perfusion, each mouse brain was isolated from the skull and fixed overnight in formaldehyde, after which each brain was dehydrated and embedded in paraffin. Next, a series of brain sections (10 μm) were cut from the paraffin-embedded tissue using a Leica microtome (Leica Microsystems, Bannockburn, IL, USA). For Nissl staining, the sections were deparaffinized with xylene, rehydrated with ethanol (EtOH) at graded decreasing concentrations from 100–70%, and finally washed with distilled water. The slides with brain sections were then subjected to Nissl staining solution using 0.1% cresyl violet acetate for 8 minutes and washed with distilled water (dH_2_O). The dead neuronal cells were then enumerated by microscopic observation.

For immunohistochemical analysis of Aβ-42 peptides, brain sections were deparaffinized with xylene, rehydrated, and pretreated for 30 min at room temperature with PBS blocking buffer containing 10% normal goat serum (Vector Laboratories Inc. Burlingame, CA, USA). The sections were then incubated with mouse anti-Aβ-42 peptide antibody (Chemicon International, Inc. Billerica, MA, USA) for 12 hr, at a dilution of 1:100 in tris-buffered saline (TBS) blocking buffer. The antigen-antibody complexes were subsequently visualized with biotinylated secondary antibody (goat anti-mouse)-conjugated HRP streptavidin (Histostain-Plus Kit; Zymed, South San Francisco, CA, USA) used at a dilution of 1:100 in TBS blocking buffer. Aβ peptides were detected using stable 3,3'-diaminobenzidine (DAB; Invitrogen, Carlsbad, CA, USA), and observed with Leica Application Suite (Leica Microsystems).

### Western and slot blotting

The total protein content of colon lysates collected from NT and Tg mice were evaluated by the Bradford’s method. Total proteins (30 μg) were separated by 4%–20% sodium dodecyl sulfate-polyacrylamide gel electrophoresis (SDS-PAGE) for 3 h, after which the resolved proteins were transferred to nitrocellulose membranes (2 h at 40 V). Each membrane was then incubated separately with following primary antibodies, overnight at 4°C: anti-mAChR M2 (Alomone Labs, Jerusalem, Israel), anti-mAChR M3 (Alomone Labs, Jerusalem, Israel), anti-PI-3K, anti-p-PI3K, anti-PKC, anti-p-PKC, eIF2α, p-eIF2α, CHOP (all from Cell Signaling Technology Inc., Cambridge, MA, USA), anti-Gα (Abcam, Cambridge, UK), IRE1α (Santa Cruz Biotechnology, Santa Cruz, CA, USA), p-IRE1α (Santa Cruz Biotechnology), Bcl2 (Abcam, Cambridge, UK), and anti-β-actin (Cell Signaling Technology Inc.). Next, the membranes were washed with washing buffer (137 mM NaCl, 2.7 mM KCl, 10 mM Na_2_HPO_4_, 2 mM KH_2_PO_4_, and 0.05% Tween 20) and incubated with horseradish peroxidase-conjugated goat anti-rabbit IgG (Zymed Laboratories, South San Francisco, CA, USA) at a dilution of 1:1,000, at room temperature for 2 h. Finally, the membrane blots were developed using Chemiluminescence Reagent Plus kits (Pfizer, New York, NY, USA and Pharmacia, New York, NY, USA).

To conduct slot blot analysis, total protein (12.5 μg) of brain tissues were transferred to a nitrocellulose membrane using a Slot Blot kit (Amersham Pharmacia Biotech, Piscataway, NJ, USA). The membrane was subsequently treated with primary rabbit anti-Aβ-42 peptide (Invitrogen, Carlsbad, CA, USA) antibody and horseradish peroxidase (HRP)-conjugated goat antirabbit IgG (Invitrogen). Finally, the membrane peptide signal was detected by the same method used in Western blot.

### Reverse transcription polymerase chain reaction (RT-PCR)

Total RNA was isolated from the frozen colons using RNAzol B solution (Tet-Test Inc., Friendswood, TX), according to the manufacturer’s protocols. cDNA was synthesized using 5 μg of RNA, and each gene was amplified by subjecting the samples to 28–32 cycles consisting of 30 s at 94°C, 30 s at 62°C and 45 s at 72°C, in a Perkin-Elmer Thermal Cycler using specific primer (Table 1). The PCR products were quantified using 1% agarose gels and a Kodak Electrophoresis Documentation and Analysis System 120.

**Table 1:** List of primer sequences.

Primer	Sequence (5’-3’)
AQP8 F	GTAGTATGGACCTACGTGAGATCAAGG
AQP8 R	AGAACCTTTCCTCTGGACTCACCACC
MUC2 F	GCTGCTCATTGAGAAGAACGATGC
MUC2 R	CTCTCCAGGTACACCATGTTACCAGG
M1 F	GCAGCAGCTCAGAGAGGTCACAG
M1 R	GATGAAGGCCAGCAGGATGG
M2 F	CCAGTATCTCCAAGTCTGGTGCAAGG
M2 R	GTTCTTGTAATGACACATGAGGAGGTGC
M3 F	GTCACTTCTGGTTCACCACCAAGAGC
M3 R	GTGTTCACCAGGACCATGATGTTGTAGG
M4 F	AGCCGCAGCCGTGTTCACAA
M4 R	TGGGTTGAGGGTTCGTGGCT
M5 F	CCCGTTGTTGAGGTGCTTCTAC
M5 R	GTCTCCGTCATGACCATACTCTA
XBP-1 F	AAACA GAGTA GCAGC ACAGA CTGC
XBP-1 R	CTTCC AGCTT GGCTG ATGAG GTCC
GADD34 F	TAGAG AGCAA GAAGT GGAGC ACACA GC
GADD34 R	CGTCA TCTTC TTCTT CTGTG TCCTC
Actin F	GTGGGGCCCCCAGGCACCAGGGC
Actin R	CTCCTTAATGTCACGCACGATTTC

### Histopathological analysis of colon

Colons collected from NT and Tg mice were fixed in 10% formalin for 12 h, embedded in paraffin wax, and then sectioned into 5 μm sections that were subjected to hematoxylin & eosin staining (H&E, Sigma-Aldrich Co., Saint Louis, MO, USA). Morphological features of these sections were observed under light microscopy, after the mucosa thickness, muscle thickness, flat luminal surface thickness, number of goblet cells, and number of crypt of Lieberkuhn were measured, using the Leica Application Suite (Leica Microsystems, Switzerland).

For mucin staining, colons collected from NT and Tg mice were fixed in 10% formalin for 48 h, embedded in paraffin wax, sectioned into 3 μm sections, and subsequently deparaffinized with xylene and rehydrated. Next, the tissue sections were rinsed with distilled water and stained with an Alcian Blue Stain kit (IHC WORLD, MD, USA). Finally, the morphological features in the stained colon sections were observed by light microscopy.

For immunohistochemical analysis of APP, the brain sections were deparaffinized with xylene, rehydrated, and pretreated for 30 min at room temperature with PBS blocking buffer containing 10% normal goat serum (Vector Laboratories Inc. Burlingame, CA, USA). The sections were then incubated with anti-APP antibody (Sigma-Aldrich Co., Saint Louis, MO, USA) at a dilution of 1:100 in Tris-buffered saline (TBS) blocking buffer for 12 h. The antigen-antibody complexes were subsequently visualized with biotinylated secondary antibody (goat anti-mouse)-conjugated HRP streptavidin (Histostain-Plus Kit; Zymed, South San Francisco, CA, USA) at a dilution of 1:100 in TBS blocking buffer. APP were detected using stable 3,3′-diaminobenzidine (DAB; Invitrogen, Carlsbad, CA, USA) and observed with Leica Application Suite (Leica Microsystems).

### Transmission electron microscopy (TEM) analysis

Colon tissues collected from 5 mice from each of the five treatment groups were fixed in 2.5% glutaraldehyde solution, rinsed with 1x PBS solution, dehydrated with ascending concentrations of EtOH solution, post-fixed in 1% osmium tetroxide (OsO_4_) for 1–2 h at room temperature, and embedded in Epon 812 media (Polysciences, Hirschberg an der Bergstrasse, Germany). Subsequently, ultra-thin sections of the colon tissue (70 nm thick) were placed on holey formvar-carbon coated grids, following which the grids were subjected to negative staining using uranyl acetate and lead citrate. Morphological features of tissues and cells were examined by using TEM (Hitachi, Tokyo, Japan).

### Treatment of agonist and antagonist in primary smooth muscle of rat intestine cells (pRISMC)

pRISMC prepared from intestines of NT and Tg infant mice were seeded into culture dishes (100 mm diameter) at a density of 10^7^ cells in 10 ml, then incubated with 1 mM arecoline hydrobromide (Sigma-Aldrich Co.) or 10 μM atropine (Sigma-Aldrich Co.) for 10 min at 37°C. Subsequently, these cells were collected by centrifugation at 848 x g for 10 min and used to measure the expression levels of specific proteins.

### Statistical analysis

A statistical significance was evaluated using One-way Analysis of Variance (ANOVA) (SPSS for Windows, Release 10.10, Standard Version, Chicago, IL, USA) followed by Tukey post hoc t-test for multiple comparison. All values were expressed as the means ± SD and a P value (P < 0.05) was considered statistically significant.

## Results

### Identification of behavioral dysfunction and damage to neuronal cells in Tg mice

**Fig 1:**
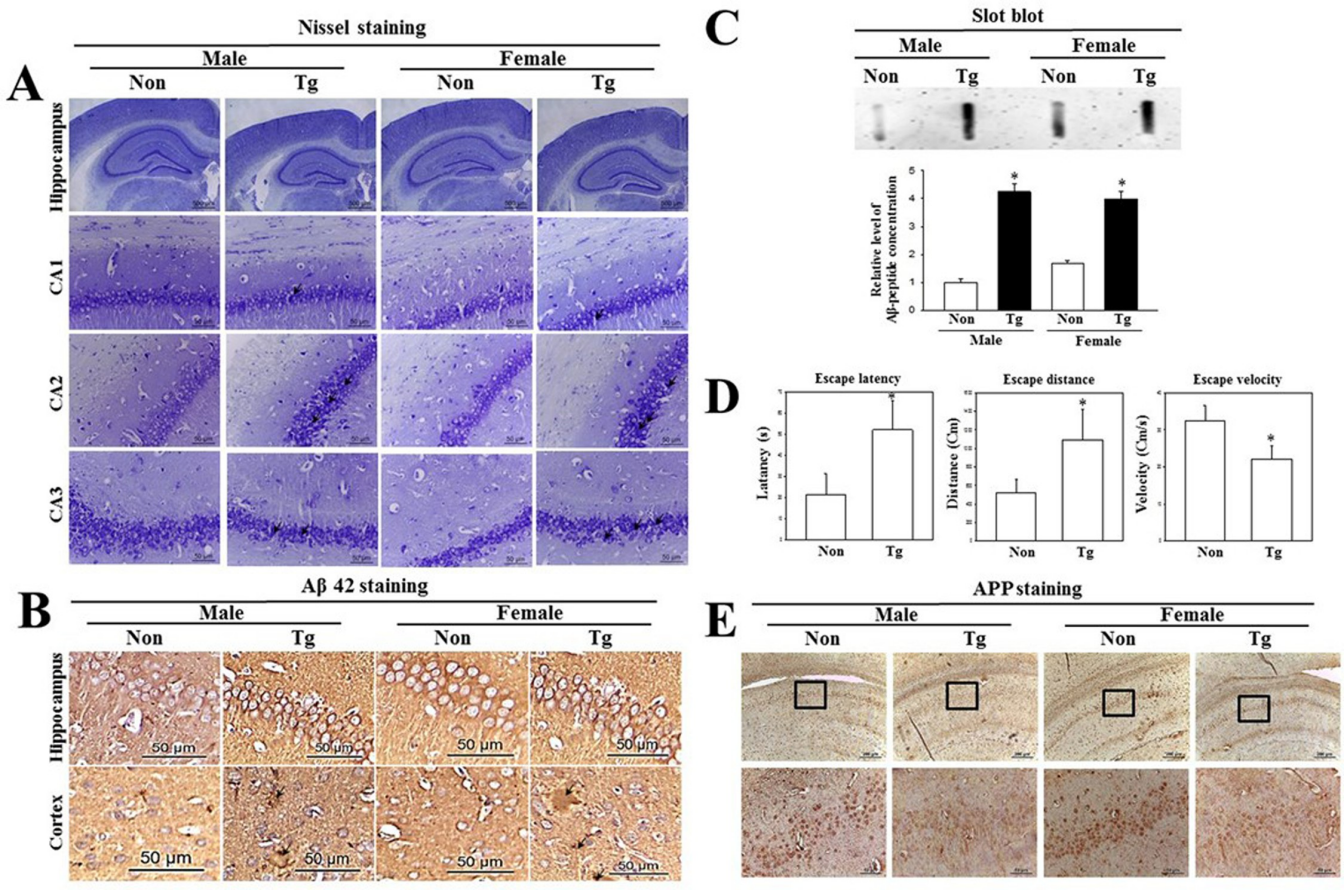
Survival of neuronal cells and deposition of Aβ peptides. Brains were collected from subset groups, and histological changes were determined as described in the Materials and Methods. (A) Slide bearing sections of brain tissue were stained with Nissl and observed at 40× or 200× magnification. The total number of neuronal cells was calculated per 80 mm^2^. (B) The accumulation of Aβ peptides in the brains of Tg mice were detected by immunohistochemical staining using specific antibody for total Aβ peptide, and their structures were observed at 400× magnification. A total of 5–6 mice were assayed per group, in triplicate, by immune staining. (C) Concentration of soluble Aβ-42 peptide was detected in brains of Tg2576 mice by slot blot analysis. (D) To test an impairment of learning and memory in the Morris water maze, the first reaching time was evaluated in the target quadrant of the pool among NT and Tg mice. (E) To detect the hippocampal neuron loss in the Tg mice, the number of neuronal cells were counted in brain sections stained with anti-APP antibodies. Data are reported as the mean ± SD. *, *p*<0.05 compared to the NT group.

One of the key indicators for pathological phenotypes of AD, elevated levels of Aβ-42 and behavioral dysfunction are reported in the brains of 9–10 month old Tg mice [34]. Hence, in order to confirm the successful enhancement of Aβ-42 production and the behavioral dysfunction, we measured the accumulation of Aβ-42 in Tg mice the memory defects and by immunohistochemical analysis and water maze test. Our results revealed that the number of dead neuronal cells was slightly higher in the granule cell layer of CA1 and CA3 in the Tg group, as compared to the NT group (Fig 1A). Also, immunohistochemical and slot blot analysis showed significantly increased levels of Aβ-42 in both male and female Tg mice as compared to the NT group, although there are some differences in their increase levels (Fig 1B and 1C). Furthermore, escape latency and distance during water maze test were remarkably increased in Tg mice compared with NT mice (Fig 1D). A significant neuronal loss was observed in the hippocampus of Tg mice (Fig 1E). Our results thus confirm that the Tg mice used in the current study show prominent pathological phenotypes for AD.

### Alterations in the feeding behavior and excretion parameters

**Fig 2:**
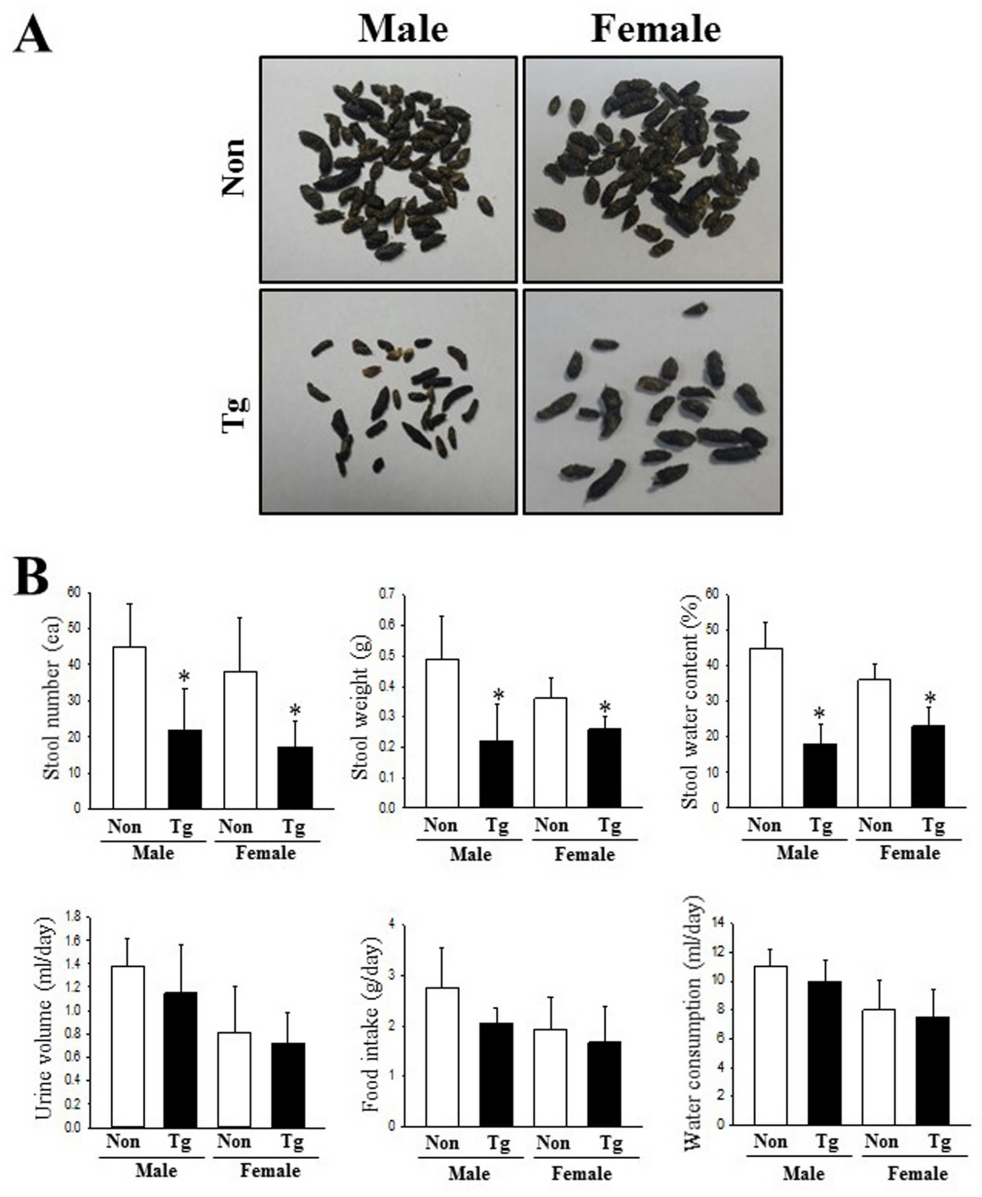
Alterations in the feeding behavior and constipation parameters of Tg mice. (A) Morphological features of stools were observed after collection and taking pictures. (B) Excretion parameters and feeding behaviors were measured in subset groups (n = 10) every morning during the experimental period, as described in Materials and Methods. Data are reported as the mean ± SD. *p<0.05 relative to the NT group.

We next examined whether the pathological phenotypes for AD affect the feeding behavior and excretion parameters of Tg mice. To achieve this, the food intake, water consumption, frequency of stools and urine volume were measured for Tg mice housed in metabolic cages. As shown in Fig 2, three related factors for feeding behavior (body weight, food intake and water consumption) did not differ significantly between the NT and Tg groups. However, the number, weight and water content of stools were significantly decreased in the Tg mice relative to those in the NT group, although the urine volume remained constant. Also, the decrease rate of these factors was greater in males than in females (Fig 2 and S1 Table). These results suggest that AD symptoms of Tg mice induce an alteration in the excretion parameters that are indicative of chronic constipation, without any significant effects on the feeding behavior.

### Histopathological alterations in the colon of Tg2576 mice

**Fig 3:**
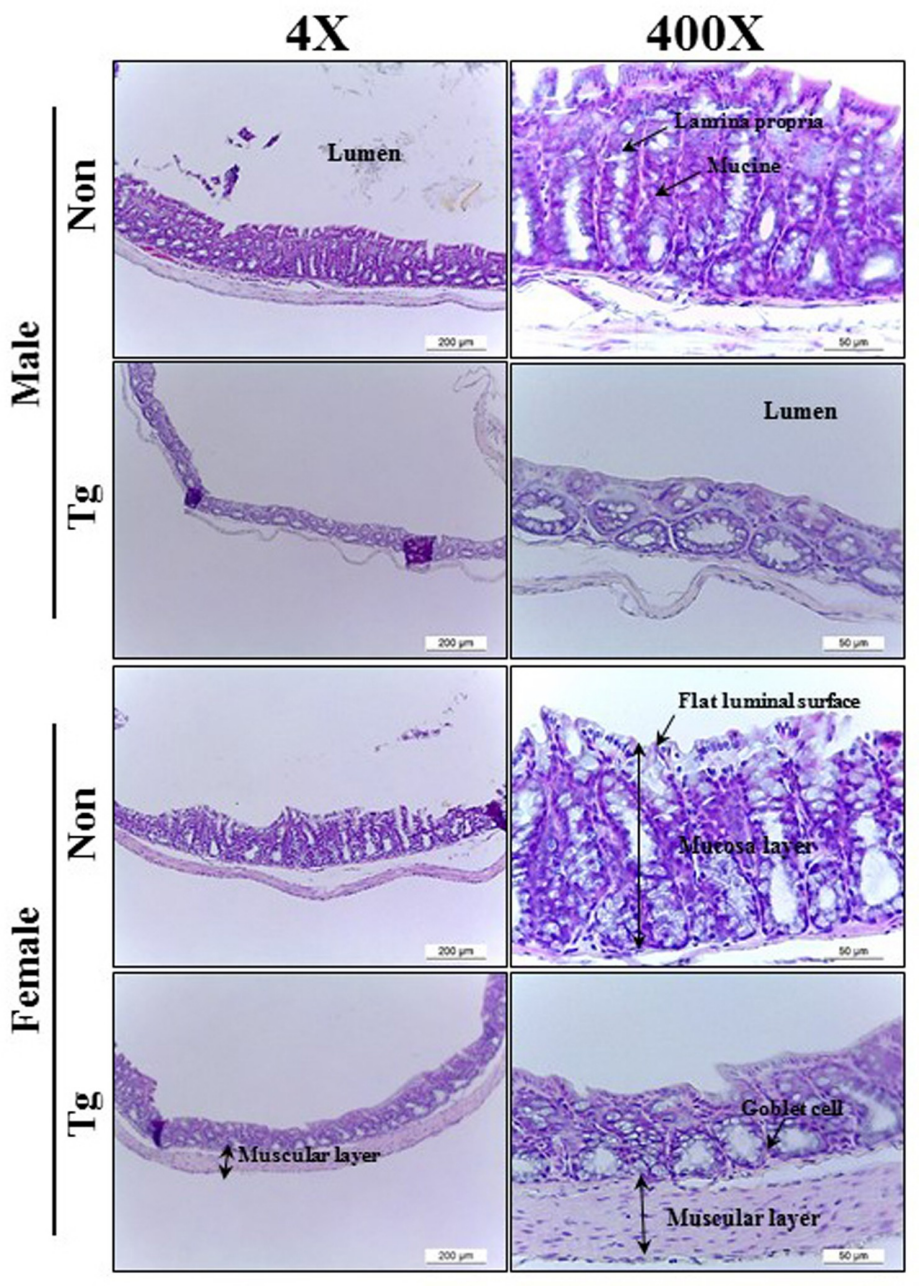
Alteration of histological structures in the colon of Tg mice. Using a light microscope, H&E stained sections of colons from the NT and Tg groups were observed at 40× and 200× magnification. Totally, 5–6 mice per group were assayed in triplicate by H&E staining.

To investigate whether the histopathological features of chronic constipation could be detected in the colon of Tg mice, we observed for changes of several histological parameters in H&E stained colon sections of Tg mice. Although histopathological changes were detected in all male and female Tg groups, wider variations of these alterations were observed in males as compared to female Tg mice. The thickness of mucosa, muscle and flat luminal surface were significantly shorter (70%, 77% and 80%, respectively) in the Tg group than in the NT group (Fig 3 and Table 2). Furthermore, the number of goblet cells and crypt of Lieberkuhn were 52% and 42% lower, respectively, in the Tg group than the NT group. Notably, the observed morphology of the crypt was spherical in the Tg group, while the slender structure was maintained in the NT group (Fig 3). The aforementioned results indicate that the pathological symptoms of AD induce alterations similar to the histopathological features of constipation in the colon of the Tg model. Furthermore, the significantly decreased mucosal thickness of Tg mice can be caused by an apoptosis-independent mode of programmed cell death in the intestinal epithelium, although apoptosis plays an important role in epithelium turnover and tissue homeostasis, as reported by van der Flier and Clevers [38] and Gunther et al. [39].

**Table 2:** Alterations in the histological parameters of Tg mice.

	Male		Female	
Categories	NT	Tg	NT	Tg
Mucosa thickness (μm)	189.2±9.2	56.2±5.7*	164.4±18.4	71.1±9.4*
Muscle thickness (μm)	48.2±3.4	10.8±1.2*	32.5±1.8	22.1±4.7*
Flat luminal surface thickness (μm)	21.2±1.3	4.2±0.8*	14.2±1.1	6.8±1.4*
Number of goblet cell (ea)	175.5±22.4	82.2±12.5*	155.7±17.4	68.9±9.8*
Number of crypt of Lieberkuhn (ea)	67.8±5.4	39.2±5.8*	52.4±7.2	40.1±6.6*

*, *p*<0.05 compared to the NT group.

### Changes on the ultrastructure of crypts in colon

**Fig 4:**
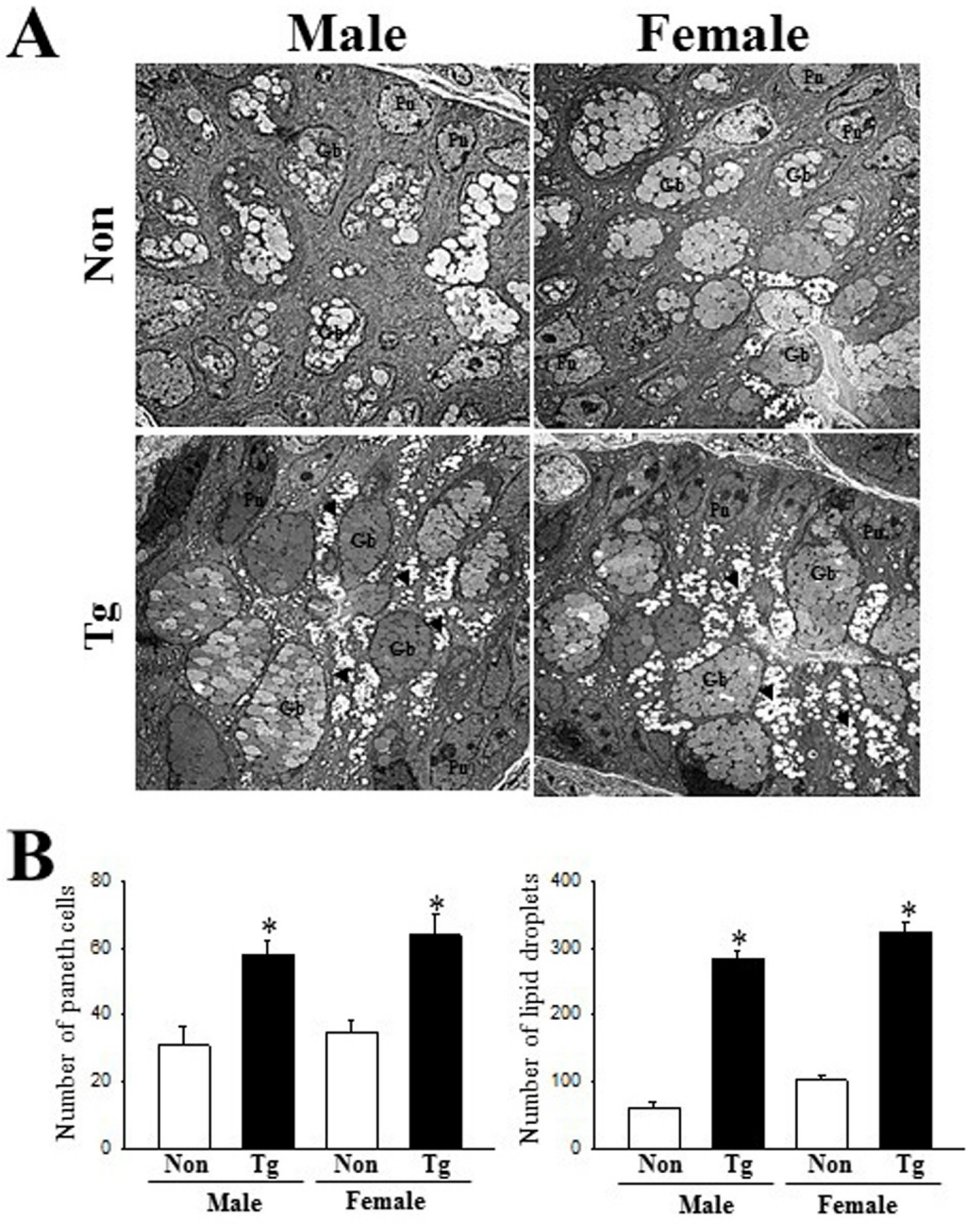
TEM images of colon. (A) Ultrastructure of the crypt in the NT and Tg mice was viewed by TEM at 1800× magnification. (B) The number of Paneth cells and lipid droplets were measured in extracellular matrix using the Leica Application Suite (Leica Microsystems, Switzerland). Five or six mice per group were assayed in triplicate by TEM analysis. Data are reported as the mean ± SD. *, *p*<0.05 compared to the NT group. Lm, lumen of crypt; Gb, goblet cells; Pn, paneth cells; Gr, granule cells; Ld, lipid droplets; SV, Secretory vesicles.

Next, we examined an alteration on the histopathological ultrastructure of colons to assess for alterations associated with constipation in Tg mice. To achieve this, we performed TEM analysis to observe the ultrastructural changes of the crypt using thin tissue sections of the colon. In the NT group, the crypt of Lieberkuhn was clearly observed as a ring structure in which a central lumen is surrounded by enterocytes, goblet cells and Paneth cells. However, the ultrastructure of the crypt was dramatically altered in the Tg group (Fig 4); we observed an increase in the number of Paneth cells, lipid droplets, and the average size of goblet cells (Fig 4B). These results indicate that AD pathological symptoms induce an alteration in the ultrastructure of the crypt, perceived as increase in the abundance of lipid droplets and Paneth cells in the crypt of the colons of Tg mice.

### Dysfunction of mucin secretion capability

**Fig 5:**
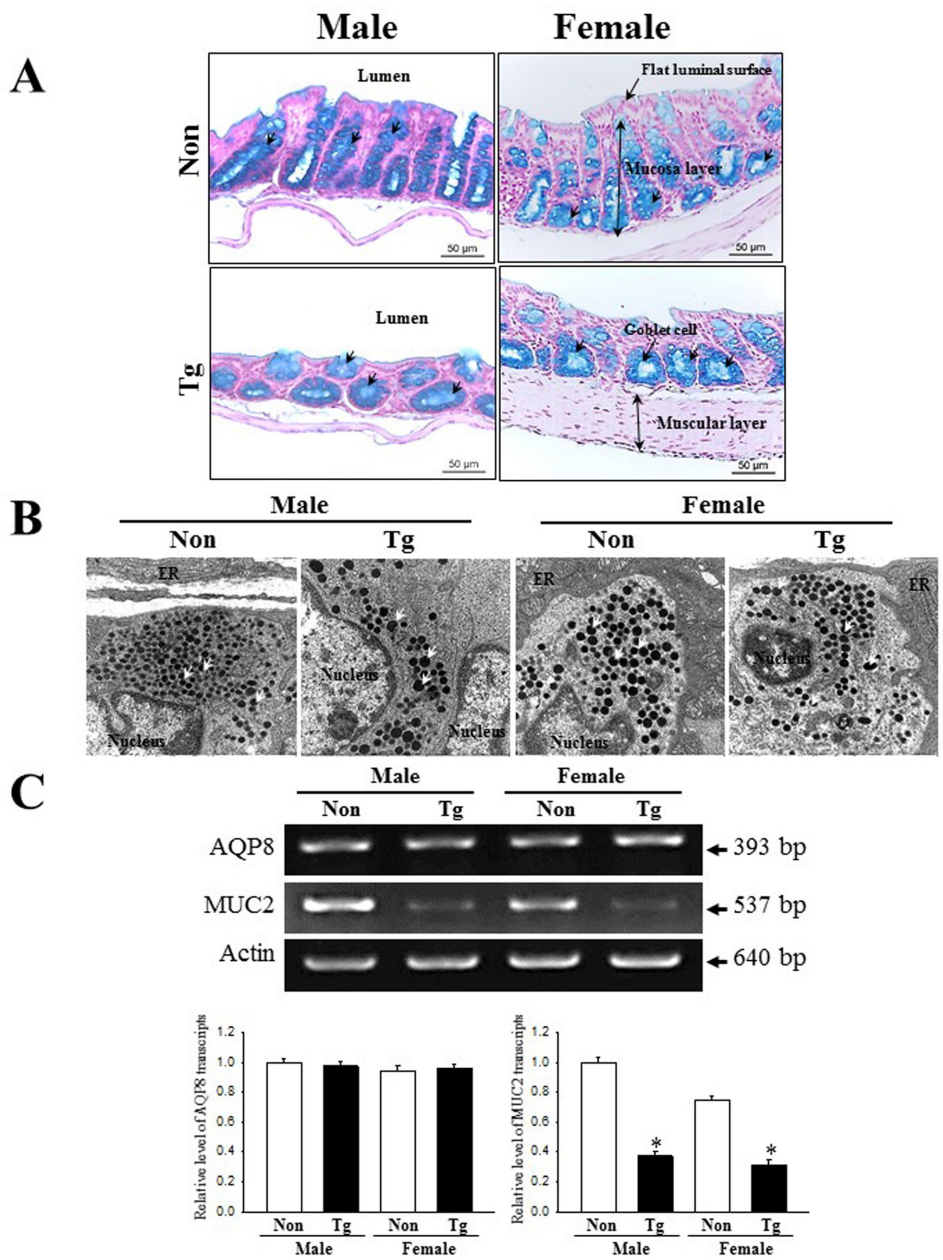
Detection of mucin secretion and membrane water channel expression. (A) Mucin secreted from the crypt layer cells was stained with alcian blue at pH 2.5. Images were observed at 100× magnification. A total of 5–6 mice per group were assayed in triplicate by alcian blue staining. (B) The distribution of secretory vesicles in Paneth cells were observed by TEM at 4,000× magnification. (C) The levels of MUC2 and AQP8 transcripts in the total mRNA of colons were measured by RT-PCR using specific primers. After the intensity of each band was determined using an imaging densitometer, the relative levels of MUC2 and AQP8 mRNA were calculated based on the intensity of actin as endogenous control. Five or six mice per group were assayed in triplicate by RT-PCR assays. Data are reported as the mean ± SD. *, *p*<0.05 compared to the NT group.

To investigate alterations in the mucin secreting capability of Tg mice, we assessed the levels of mucin in colon tissue sections of the subset group, stained with Alcian blue. The dark blue stained regions were concentrated in the crypts of the colon mucosal layers of both Tg and NT groups. The total level of mucin was lower in the Tg group than the NT group, although some differences were observed between male and female genders (Fig 5A). Additionally, the alteration of mucin secretion was entirely reflected in the distribution of secretory vesicles. The number of secretory vesicles were significantly decreased in the Paneth cells of male Tg mice, but similar levels were maintained in female Tg mice (Fig 5B). Furthermore, a similar pattern was detected in the expression levels of MUC2 mRNA; the transcription level of this gene was significantly decreased in the Tg group as compared to the NT group (62% and 71%, respectively).

We also investigated whether mucin secretion induced by AD pathological symptoms is accompanied by alteration in the expression of a membrane water channel by assessing the level of AQP8 mRNA in the colon of subset groups. We observed that the level of AQP8 mRNA was consistently maintained in both Tg and NT mice (Fig 5C). Taken together, these results indicate that dysfunction of the mucin secretion capability in the colon is linked with pathological symptoms of AD.

### Dysregulation of the mAChRs downstream signaling pathway

**Fig 6:**
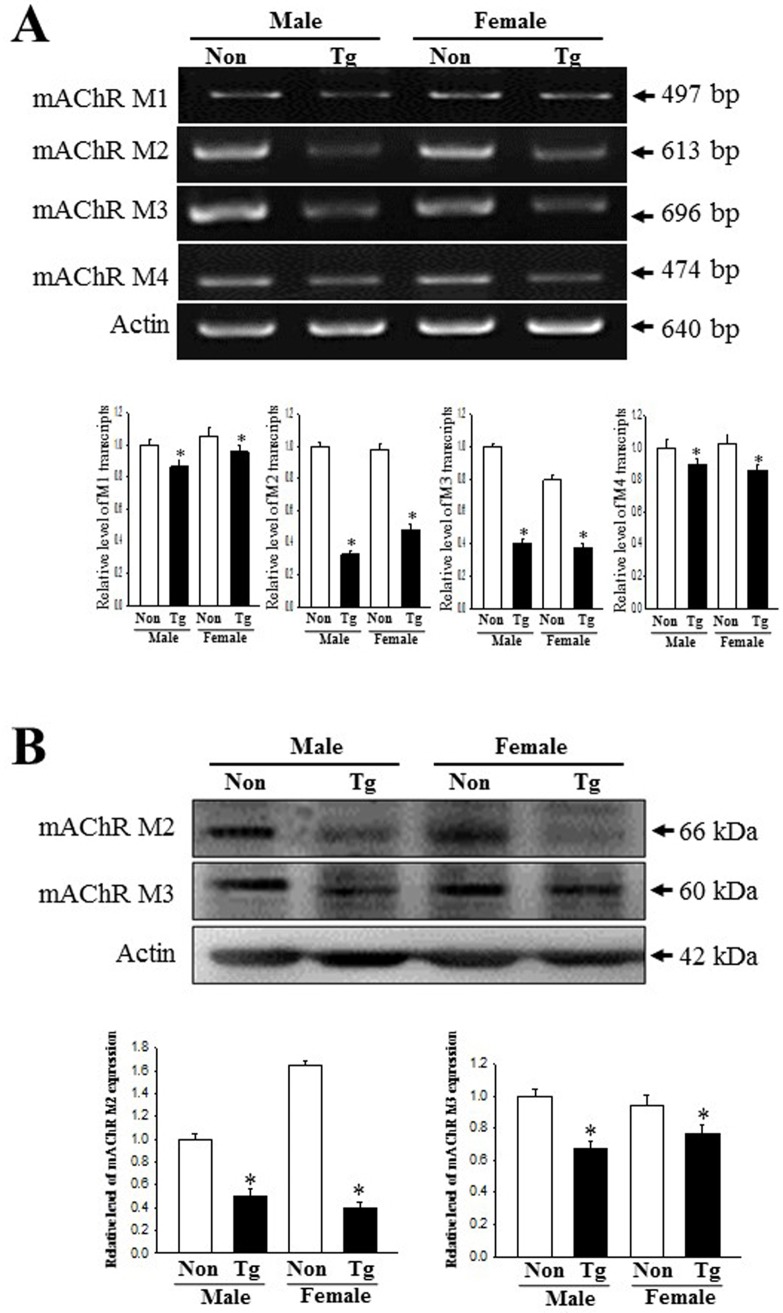
Expression of the mAChRs transcripts and proteins. (A) Changes in the mRNA level of the mAChR M1, 2, 3 and 4 in the NT and Tg mice. The transcription level of the mAChR M2 and M3 gene were examined by RT‐PCR using specific primers. (B) Expression of mAChR M2 and 3 proteins. After the preparation of colon homogenates, two proteins were measured by Western blotting using HRP-labeled anti-rabbit IgG antibody. The intensity of each band was determined using an imaging densitometer. The relative levels of these proteins were calculated, based on the intensity of actin protein. A total of 5–6 mice per group were assayed in triplicate by Western blotting. Data are reported as the mean ± SD. *, *p*<0.05 compared to the NT group.

**Fig 7:**
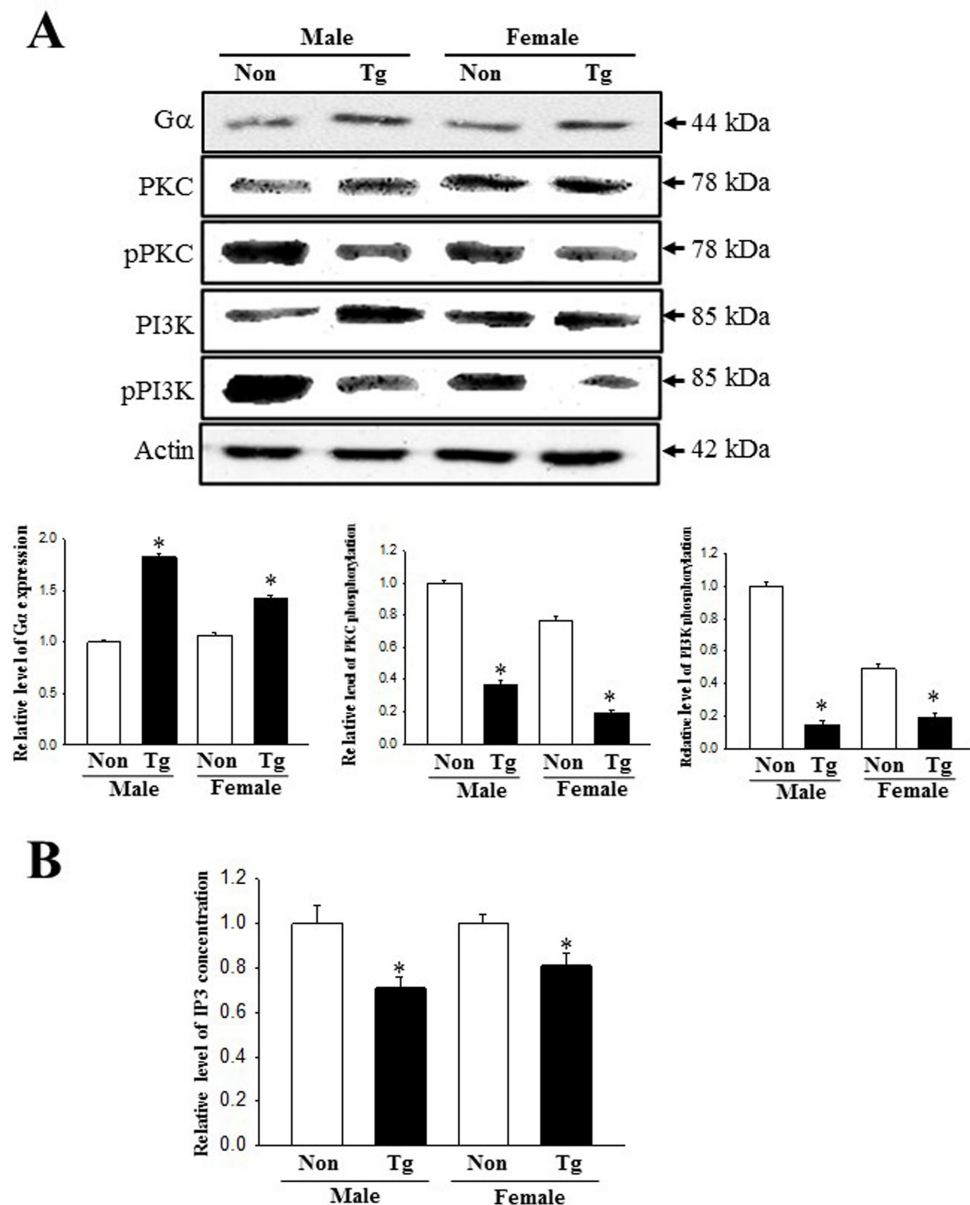
Expression of key components in the mAChR M2 and M3 downstream signaling pathway. (A) Alterations in the phosphorylation of PKC and PI3K proteins. Expression levels of PKC, p-PKC, PI3K and p-PI3K protein were determined by Western blot analysis using HRP-labeled anti-rabbit IgG antibody. Band intensities were determined using an imaging densitometer, and the expressions of the six proteins were calculated relative to the intensity of actin. (B) Alteration on the IP3 concentration. The tissue homogenate used to measure the IP3 concentration was prepared from colons collected from Tg mice. The IP3 concentration was quantified by an enzyme-linked immunosorbent assay (ELISA) with a sensitivity of 0.39 ng/ml and an inter-assay coefficient of variation of 1.56–100 ng/ml. Total 5–6 mice per group were analyzed in triplicate by Western blot. Data are reported as the mean ± SD. * indicates *p* < 0.05 compared to the NT group.

To investigate whether the constipation phenotypes of Tg mice are associated with the dysregulation of the mAChRs and their downstream signaling pathway, we measured the expression of mAChRs and their downstream members in the colon of the subset groups. The transcripts levels of four type mAChRs (M1-4) were lower in Tg mice than NT group although the decrease rate was greater in mAChR M2 and M3 compared with mAChR M1 and M4 (Fig 6A). Also, the alteration of mAChR M2 and M3 transcripts level with great decrease rate were completely reflected in the protein level of these (Fig 6B). Furthermore, several mAChR downstream members showed a similar pattern of expression for all experimental groups. A decreased response in mAChRs expression was detected in the phosphorylation levels of PKC and PI3K, members of the downstream pathway of mAChR M2 and M3, respectively (Fig 7A). The IP3 concentration decreased by 29% and 18% in male and female Tg group relative to the NT group (Fig 7B). However, the expression of the Gα protein (one of mAChR-coupled proteins with phospholipase C (PLC) activation ability) was significantly increased in the Tg group as compared to the NT group (83% and 45%, respectively)(Fig 7A). These patterns were maintained in male and female group. These results indicate that the constipation phenotypes detected in Tg mice correlate with the expression levels of mAChRs and their downstream signals in the colons.

### Difference response to mAChR agonist and antagoinst in pRISMC

**Fig 8:**
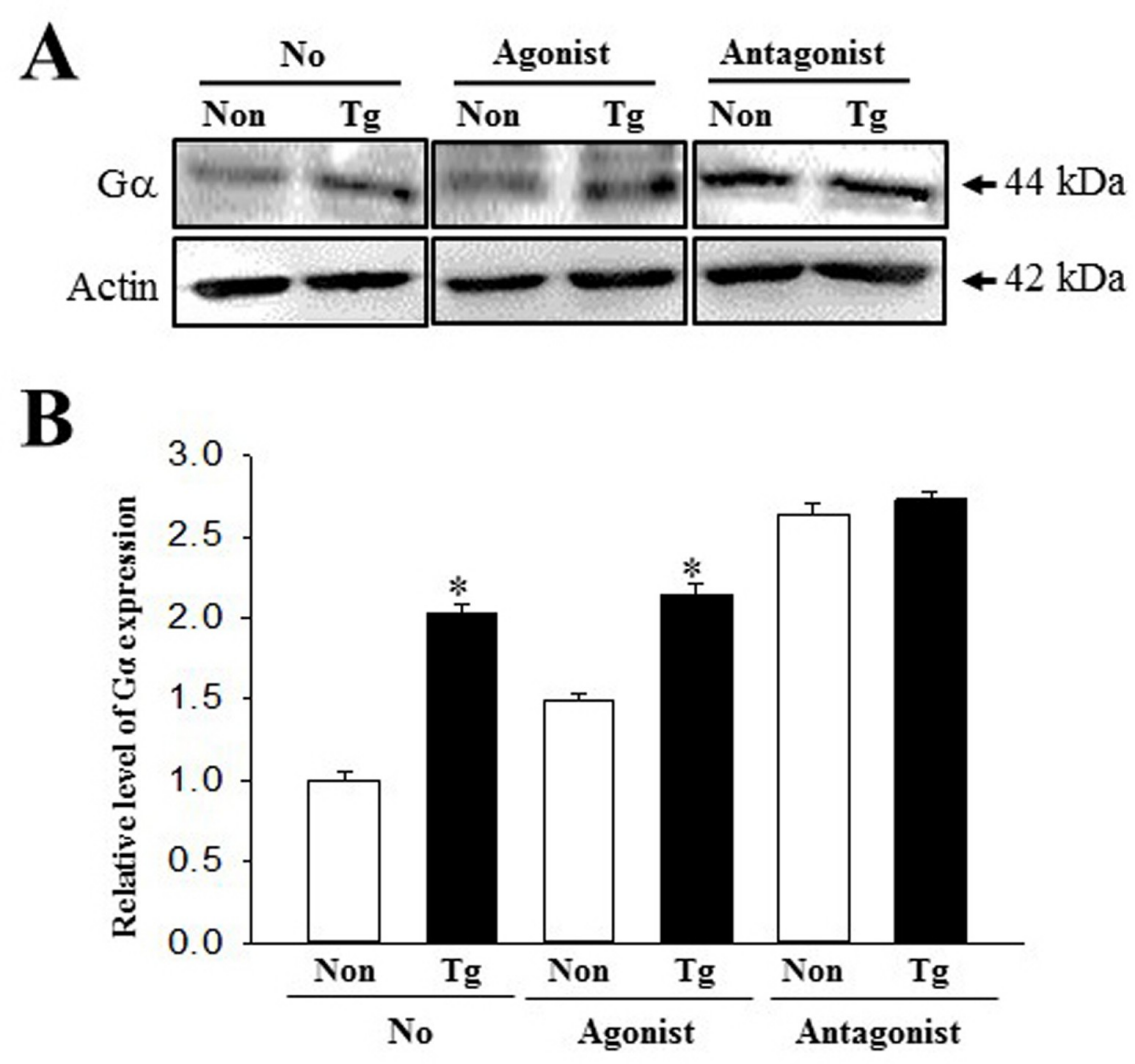
Expression of Gα protein in pRSMC treated with agonist and antagonist. After the preparation of cells lysates, two proteins were measured by Western blotting using HRP-labeled anti-rabbit IgG antibody. The intensity of each band was determined using an imaging densitometer. The relative levels of Gα protein were calculated, based on the intensity of actin protein. Three to four mice were used to collect pRSMC and three culture dishes were assayed in duplicate by Western blotting. Data are reported as the mean ± SD. *, p<0.05 compared to the NT group.

To conform the correlation between mAChR and constipation in Tg mice, alterations in the mAChR downstream signaling pathway were measured in pRISMCs treated with agonist and antagonist. The expression levels of Gα protein were higher with 103% in the Vehicle-treated Tg group compared with the Vehicle-treated NT group. These patterns were maintained in agonist-treated group. However, these alterations were completely disappeared after the treatment of antagonist (Fig 8B). The present results indicated that mAChR signaling pathway may tightly linked with induction of constipation in Tg mice.

### Dysregulation of the ER stress response

**Fig 9:**
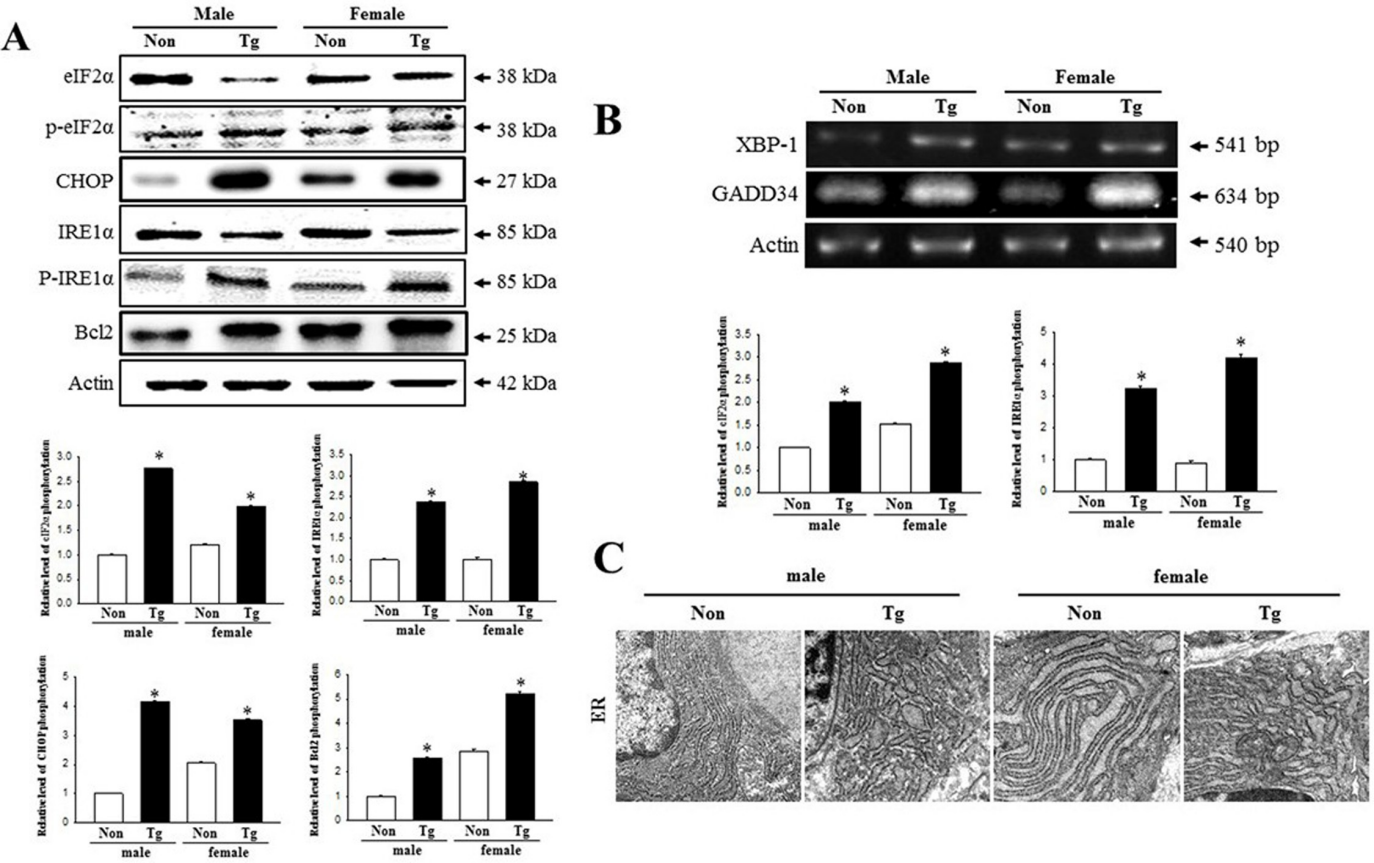
Detection of ER stress response. (A) Expression of related proteins, including eIF2α, p-eIF2α, CHOP, IRE1α, p-IRE1α, and Bcl-2, were measured by Western blotting using HRP-labeled anti-rabbit IgG antibody. After the intensity of each band was determined using an imaging densitometer, the relative levels of the six proteins were calculated based on the intensity of actin protein. Five or six mice per group were assayed in triplicate by Western blotting. (B) Changes in the mRNA level of the XBP-1 and GADD34 in the NT and Tg mice. The transcription level of the XBP-1 and GADD34 gene were examined by RT‐PCR using specific primers. Data are reported as the mean ± SD. *, *p*<0.05 compared to the NT mice. (C) Ultrastructure of the ER in Paneth cells was detected by TEM at 4,000× magnification. N, nucleus; ER, endoplasmic reticulum.

Finally, our investigation to assess whether constipation of Tg mice is associated with the dysregulation of the ER stress response revealed an alteration in the expression of ER stress related proteins and ER structure when comparing the colons of the Tg mice and NT mice. The levels of eIF2α phosphorylation and IRE1α were enhanced with an average 228.5% and 266.5%, respectively, in Tg mice compared to NT mice, although there were gender differences. Also, a similar level of increase was detected for CHOP and Bcl-2 proteins in Tg mice (Fig 9A). Furthermore, the transcript levels of XBP-1 and GADD34 were higher (270% and 345%, respectively) in Tg mice than in NT mice (Fig 9B). Similar significant alterations were also observed in the ER structure of Paneth cells. NT mice showed a stacked conformation of ER membrane sheets around the nuclear membrane; this structure was rapidly modified in the Tg mice into an irregularly arranged tubular structure with extensive swelling and significant expansion (Fig 8C). These results suggest that the constipation phenotypes detected in Tg mice correlate with the dysregulation of ER stress responses in the colons.

## Discussion

AD is the most common form of dementia in elderly people, having an estimated worldwide incidence of 35.6 million patients in 2010, which is projected to nearly double every 20 years [40]. Patients suffering from dementia often have accompanying chronic diseases. An inverse epidemiological correlation between AD and cancer has been revealed in multiple ethnic groups [41]. Diabetic patients (type 2) have a higher risk of eventually developing AD or other dementias [42]. Recent researches are therefore rapidly increasing to identify novel evidences for correlations between AD and other diseases. As part of our studies using the Tg mice model for AD, we investigated whether the AD pathological condition affects the gastrointestinal function and physiology. Although the current results provide initial clues about the association between two chronic diseases, further studies are required to investigate the relevant mechanism of action.

Several gastrointestinal (GI) motor symptoms, including excessive drooling, dysphagia, early satiety, nausea, constipation and defecatory dysfunction, are prominent and disabling manifestations of PD [43–45]. Among these symptoms, constipation is the most common GI dysfunction, having a prevalence of 60–80% in PD patients [11]. Also, rat PD models induced by microinjecting 6-hydroxydopamine (6-OHDA) display a significant alteration in complete loss of dopaminergic terminal and cell body in the striatum and *substantia nigra pars* compacta (SNC), as well as in daily fecal outputs such as fecal weight (55–60%), water content (44.5%) and stomach residual percentage (70.9%) [46, 47]. The current study measured several excretion parameters to confirm the symptoms of constipation in 11-month old Tg mice. Considering parameters such as number, weight and water content of stools observed in the 6-OHDA injected model, we obtained similar results in Tg mice, although the urine volume and food intake remained constant in our study. The present study therefore shows novel evidences that the AD model with Aβ peptide accumulation encompasses associated constipation.

Various animal models play a crucial role in developing therapeutic drugs and understanding the pathological mechanism of the disease [48, 49]. Of these models, the Tg2576 mice used in this study were developed by microinjecting the human APP695 gene containing the Swedish type mutation (K670N, M671L) under control of a hamster prion protein, into B6SJLF2 fertilized eggs. These models demonstrate AD patient-like phenotypes at 9–10 months of age, including behavioral defects, a 14-fold increase in Aβ-42 peptide levels, and formation of numerous amyloid plaques [34]. Furthermore, they also correlate with other pathological conditions or chronic diseases such as neuroinflammation, loss of noradrenergic neurons, obesity, insulin resistance and aortic atherosclerosis [50–53]. However, there are currently no scientific evidences to correlate the association between AD pathological condition and bowel dysfunction using Tg mice with AD patient-like phenotypes. Therefore, we believe that our study provides the first evidence for a possibility that constipation is induced with progressing AD in the Tg2576 mice model.

Numerous studies have presented the correlation of bowel dysfunction with PD and SCI. In case of 6-OHDA-induced PD, the daily fecal weight and water contents decreased significantly in the treated rats, although their levels ranged widely between 22–60% [46, 47, 54]. Additionally, this model showed gastric emptying delay, increased gastrointestinal tyrosine hydroxylase (TH), and decreased neuronal nitric oxide synthase (nNOS) [46, 54]. Furthermore, constipation, fecal incontinence, urinary retention and other complications are also associated with spinal cord injuries (SCI), which is an irreversible loss of sensory functions and voluntary motor control below the level of injury [55–57]. However, evidences for induction and mechanism of constipation in conditions of pathological AD have not been reported, although this disease is considered one of the neurological diseases affecting the central nerve system. In our study, we observed significant alterations in the excretion parameters, as well as in the histopathological structure, capability of mucin secretion, mAChR signaling pathway, and ER stress response in the colon, in Tg2576 mice showing AD pathological features. Although most results on excretion parameters are consistent with previous results, the present study further investigated mucin secretion, mAChR downstream signaling pathway and ER stress response to verify the mechanism of action.

mAChRs participate in the functional regulation of gut epithelial cells, which in turn regulate the process of food degradation and uptake of nutrients, electrolytes and water through a combination of acetylcholine molecules [58]. Only few studies have investigated that the mAChRs signaling pathway is linked to constipation in loperamide (Lop) treated Sprague Dawley (SD) rats. The expression of mAChR M2/M3 as well as the signaling process of their downstream members were significantly altered in the colon of constipation model, although these alterations were restored after administering several laxatives [59–61]. In the current study, a similar pathological alteration is observed in the mAChRs downstream signaling pathway, in the colon of Tg mice. These results agree with previous studies, in which the mAChRs downstream signaling pathway is shown to be disrupted under the influence of constipation. Furthermore, several mAChRs are implicated in the degeneration of cholinergic neurons and cholinergic hypofunction of AD [19]. Stimulation of mAChR M1 by agonist treatment induces the production of APPs, upregulates BACE, and activates α-secretase through the activation of PKC and MAPK, resulting in the decreasing Aβ production in various cell lines [62–65]. In the current study, the phosphorylation of PKC was lower in the colons of Tg2576 mice than in non-Tg mice. Although our findings concerning the correlation between PKC activation and AD pathogenesis do not accurately reflect previous researches, the present results provide additional evidences that the dysregulation of mAChR downstream signaling pathway may be involved as an important mechanism in constipation associated with neurodegenerative disorders. In addition, the regulation of gastrointestinal hormones can be considered as another mechanism that plays a role in constipation, since these hormones (including gastrin, motilin and somatostatin) regulate the secretion of gastric juice, constriction of gastrointestinal smooth muscles, the movement of gastrointestinal contents, and the excitation of gastrointestinal motility. Further researches are required to confirm this mechanism [66, 67].

ER stress is induced under numerous conditions related with the interruption of ER functions, such as pathological conditions, intracellular metabolic changes, abnormal diet, microenvironmental conditions, medicine treatment and administration of natural product. This correlation between ER stress and constipation has only recently been investigated [68–70]. These processes are accompanied by accumulation of unfolded or misfolded proteins as well as alterations of the ER structure [71]. The phosphorylation level of proteins indicating ER stress response significantly increases in the colon of Lop induced constipation model [69, 70]. Also, alterations of ER morphology have been reported for several pathological conditions. Dilation of ER lumen and increase of lumen density were detected in dry eye-induced mice [72], and swelling and degranulation of rough ER and expansion ER lumen were found in homocysteine-induced hepar injury with ER stress [73]. Furthermore, the expansion, swelling and fragmentation of ER has been detected in liver senescence [74] and pancreatic atrophy [75]. The response to ER stress was largely different for each AD mouse model, although they all display AD-related pathologies such as Aβ accumulation and tau hyperphosphorylation [76]. Among these models, Tg2576 mice showed a constant level of ER stress markers such as CHOP, PDI, eIF2α and GRP78 in the brain tissue, regardless of their age [76, 77]. However, these results on the ER stress responses were different from the observations of the current study. As presented Fig 7, the phosphorylation of eIFα and IRE1α were significantly enhanced in the colon of Tg2576 mice. The differences between studies can be attributed to different target organs used for analysis of ER stress response.

The present study observed some correlations between the dysregulation on the ER stress protein and ER structure, and pathological condition of the AD model, as shown in Fig 9. This is similar to previous reports wherein the ER stress responses, including ER marker proteins and ER structure, were triggered by numerous pathological insults. We believe the results of the present study provide first evidences that constipation of Tg mice showing AD phenotypes can be linked to disturbances of the ER stress response.

## Conclusions

The results of the present study suggest that the AD pathological condition induces constipation through alteration of the excretion parameters as well as changes in the histological structure, mucin secretion, mAChRs downstream signaling pathway and ER stress response of the colon. The results of the present study suggest that the AD pathological condition induces constipation through alteration of the excretion parameters as well as changes in the histological structure, mucin secretion, mAChRs downstream signaling pathway and ER stress response of the colon. These results also demonstrate that constipation should be considered an important symptom of the AD and must be taken into acount for any potential treatment.

## Supporting information

S1 Fig Structure of APPsw gene and identification of transgenes. (A) Tg2576 mice have the mutant human APP gene 695 amino acid isoform and with a double mutation (Lys670→Asn and Met671→Leu). (B) DNA-PCR analysis were performed on genomic DNA isolated from the tail of founder mouse, and the 442 bp of products were shown in Tg mice carrying the APPsw transgenes.
(TIF)

S1 Table Number of weight of stools of Tg2576 mice. (DOCX)
