# Expression of Concern: Constipation in Tg2576 mice model for Alzheimer’s disease associated with dysregulation of mechanism involving the mAChR signaling pathway and ER stress response

**DOI:** 10.1371/journal.pone.0356955

**Published:** 2026-08-26

**Authors:** The PLOS One Editors

After this article [1] was published, concerns were raised regarding Figs 1-2, 4-6 and S1 Fig. Specifically:

- In Fig 1B, the Male and Female Hippocampus Tg panels appear similar to each other.
- In Fig 2A of [1], the Female Non panel appears similar to the No panel in Fig 1B of [2].
- In Fig 4A of [1], the Male Tg panel appears similar to the Lop+Vehicle panel of the corrected Fig 4A in [3–4].
- The Actin panels in Figs 5C and 6A appear similar to each other.
- In S1B Fig, there appears to be vertical discontinuities between lanes 16 and 17 of both Male and Female panels.

The corresponding author stated that the Female Tg Hippocampus panel in Fig 1B, the Female Non panel in Fig 2A and all panels in Fig 4A are incorrect, and provided corrected versions of Figs 1, 2 and 4 with correct panels from the original experiments. They further stated that the PCR experiments shown in Figs 5C and 6A were conducted simultaneously and used the same Actin controls. They also stated that in S1B Fig, the number of samples exceeded the capacity of the gel and therefore two separate gels were run, and these runs were then arranged into a single figure. An updated S1 Fig, and corresponding caption, with the splice lines clearly indicated with white vertical lines has been provided with this notice. The original images underlying all panels in Figs 1A-B, 2A, 4A, 5C, 6A and S1B Fig have been included with this notice as S1 File.

The raw data underlying Figs 1-2, 4–9 and S1 Fig are missing from the list of Supporting Information. The authors have provided the data as S2 File. With this notice, all relevant data are now provided.

In light of the above concerns, the *PLOS One* Editors no longer have confidence in the reliability of the results presented in this article and issue this Expression of Concern. Readers are advised to interpret the article with caution.

The Female Non panel of Fig 2A in [1] was previously published in [2] under a CC BY license. No changes were made to the re-used content. The Female Non panel of Fig 2A is subject to the license that applies to the original article; please provide due attribution to the original publication when referring to this content.

**Fig 1:**
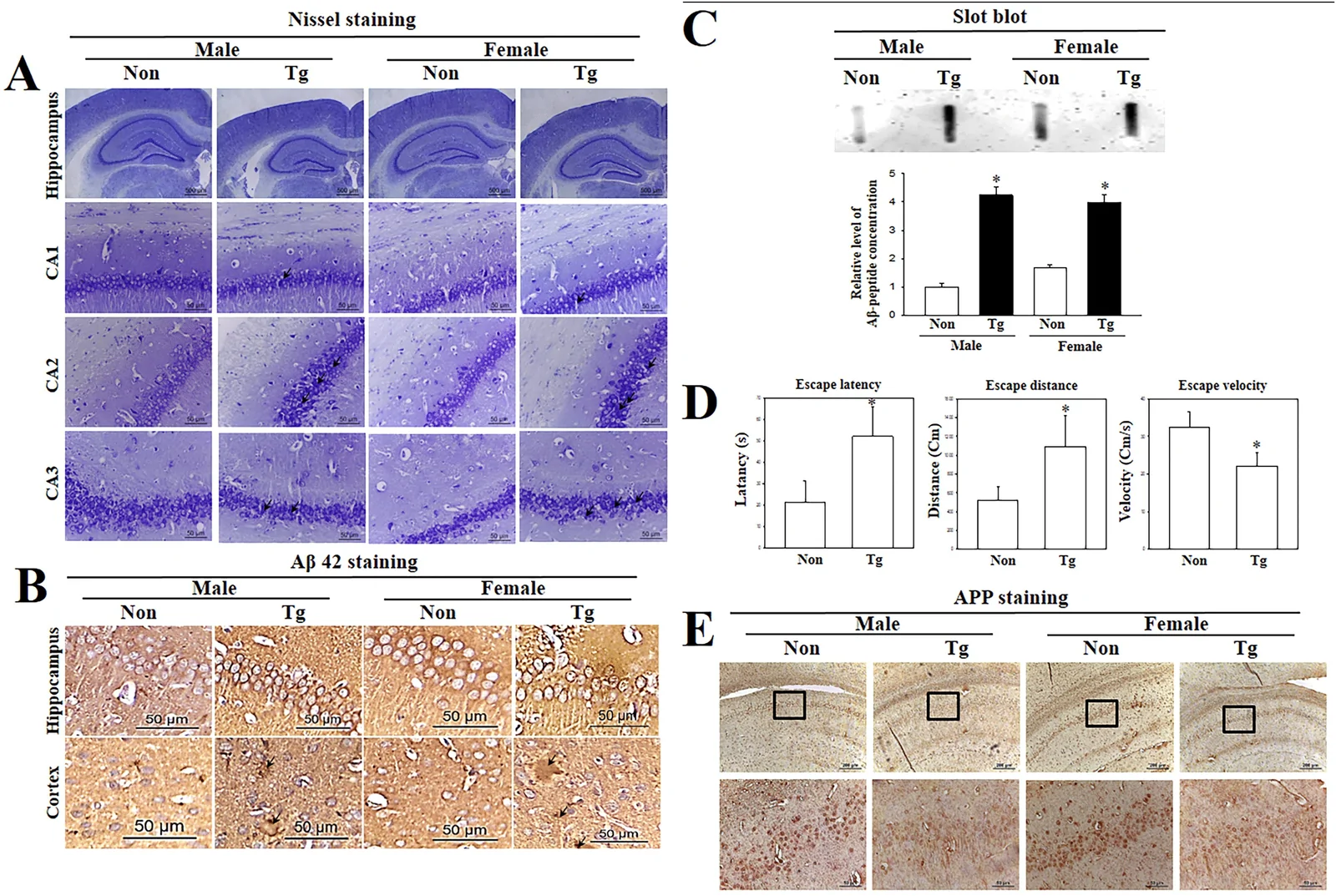
Survival of neuronal cells and deposition of Aβ peptides. Brains were collected from subset groups, and histological changes were determined as described in the Materials and Methods. (A) Slide bearing sections of brain tissue were stained with Nissl and observed at 40× or 200 × magnification. The total number of neuronal cells was calculated per 80 mm^2^. (B) The accumulation of Aβ peptides in the brains of Tg mice were detected by immunohistochemical staining using specific antibody for total Aβ peptide, and their structures were observed at 400 × magnification. A total of 5–6 mice were assayed per group, in triplicate, by immune staining. (C) Concentration of soluble Aβ-42 peptide was detected in brains of Tg2576 mice by slot blot analysis. (D) To test an impairment of learning and memory in the Morris water maze, the first reaching time was evaluated in the target quadrant of the pool among NT and Tg mice. (E) To detect the hippocampal neuron loss in the Tg mice, the number of neuronal cells were counted in brain sections stained with anti-APP antibodies. Data are reported as the mean ± SD. *, *p* < 0.05 compared to the NT group.

**Fig 2:**
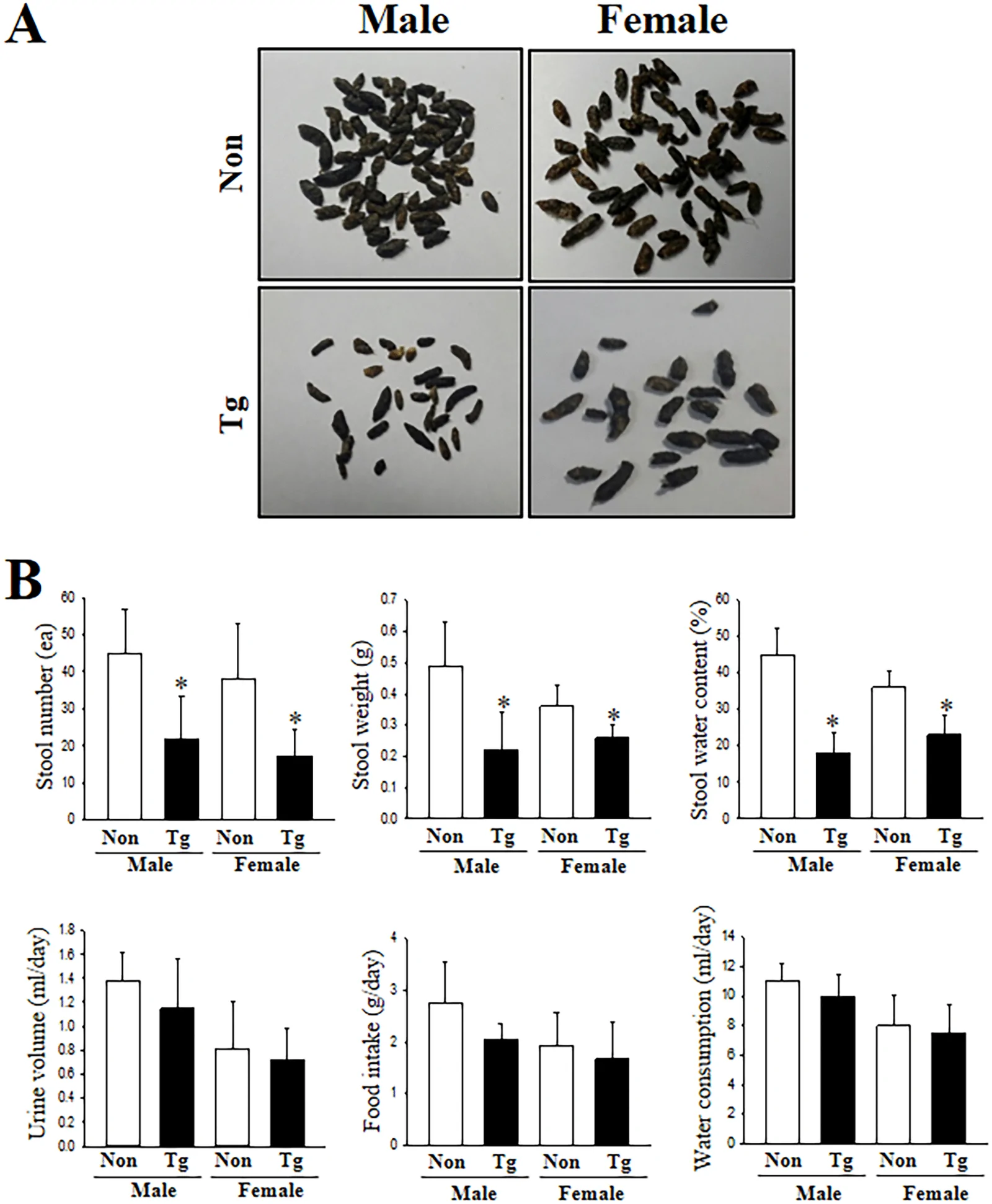
Alterations in the feeding behavior and constipation parameters of Tg mice. (A) Morphological features of stools were observed after collection and taking pictures. (B) Excretion parameters and feeding behaviors were measured in subset groups (n = 10) every morning during the experimental period, as described in Materials and Methods. Data are reported as the mean ± SD. *p < 0.05 relative to the NT group.

**Fig 4:**
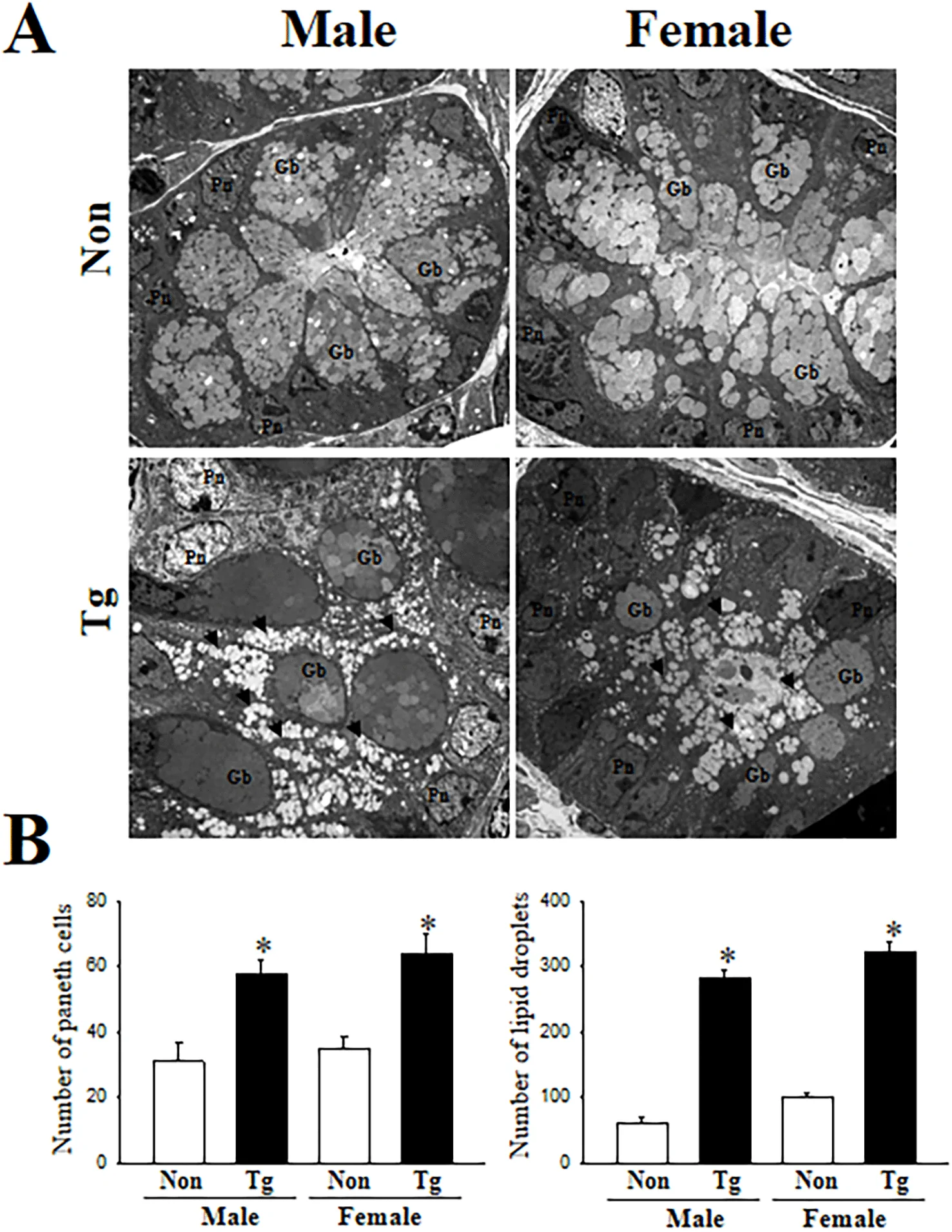
TEM images of colon. (A) Ultrastructure of the crypt in the NT and Tg mice was viewed by TEM at 1800 × magnification. (B) The number of Paneth cells and lipid droplets were measured in extracellular matrix using the Leica Application Suite (Leica Microsystems, Switzerland). Five or six mice per group were assayed in triplicate by TEM analysis. Data are reported as the mean ± SD. *, *p* < 0.05 compared to the NT group. Lm, lumen of crypt; Gb, goblet cells; Pn, paneth cells; Gr, granule cells; Ld, lipid droplets; SV, Secretory vesicles.

## Supporting information

S1 Fig Structure of APPsw gene and identification of transgenes. (A) Tg2576 mice have the mutant human APP gene 695 amino acid isoform and with a double mutation (Lys670 → Asn and Met671 → Leu). (B) DNA-PCR analysis were performed on genomic DNA isolated from the tail of founder mouse, and the 442 bp of products were shown in Tg mice carrying the APPsw transgenes. Splice lines have been indicated with vertical white lines.
(TIF)

S1 File Original images underlying all panels in Figs 1A-B, 2A, 5C, 6A and S1B Fig. Original images underlying partial panels in Figs 7A, 8A and 9A. Original and replicate images from the time of the original experiments underlying all panels in Fig 4A.
(ZIP)

S2 File Original quantitative data underlying all figures. (ZIP)

## References

[1] Kim JE, Park JJ, Lee MR, Choi JY, Song BR, Park JW, et al. Constipation in Tg2576 mice model for Alzheimer’s disease associated with dysregulation of mechanism involving the mAChR signaling pathway and ER stress response. PLoS One. 2019;14(4):e0215205. doi: 10.1371/journal.pone.0215205 30978260 PMC6461235

[2] Kim JE, Park JW, Kang MJ, Choi HJ, Bae SJ, Choi Y, et al. Laxative Effect of Spicatoside A by Cholinergic Regulation of Enteric Nerve in Loperamide-Induced Constipation: ICR Mice Model. Molecules. 2019;24(5):896. doi: 10.3390/molecules24050896 30836659 PMC6429089

[3] Kim JE, Park JW, Kang MJ, Choi HJ, Bae SJ, Choi YS, et al. Anti-Inflammatory Response and Muscarinic Cholinergic Regulation during the Laxative Effect of *Asparagus cochinchinensis* in Loperamide-Induced Constipation of SD Rats. Int J Mol Sci. 2019;20(4):946. doi: 10.3390/ijms20040946 30795644 PMC6412595

[4] Kim JE, Park JW, Kang MJ, Choi HJ, Bae SJ, Choi YS. Correction: Kim *et al*. Anti-inflammatory response and muscarinic cholinergic regulation during the laxative effect of *Asparagus cochinchinensis* in loperamide-induced constipation of SD rats. Int J Mol Sci. 2026;27(5):2228. doi: 10.3390/ijms27052228 41828734 PMC12984967

