# Supplementary figures and images for: Constipation in Tg2576 mice model for Alzheimer’s disease associated with dysregulation of mechanism involving the mAChR signaling pathway and ER stress response
Source: PLoS One. 2019 Apr 12;14(4):e0215205. doi: 10.1371/journal.pone.0215205 (PMC6461235; doi:10.1371/journal.pone.0215205)

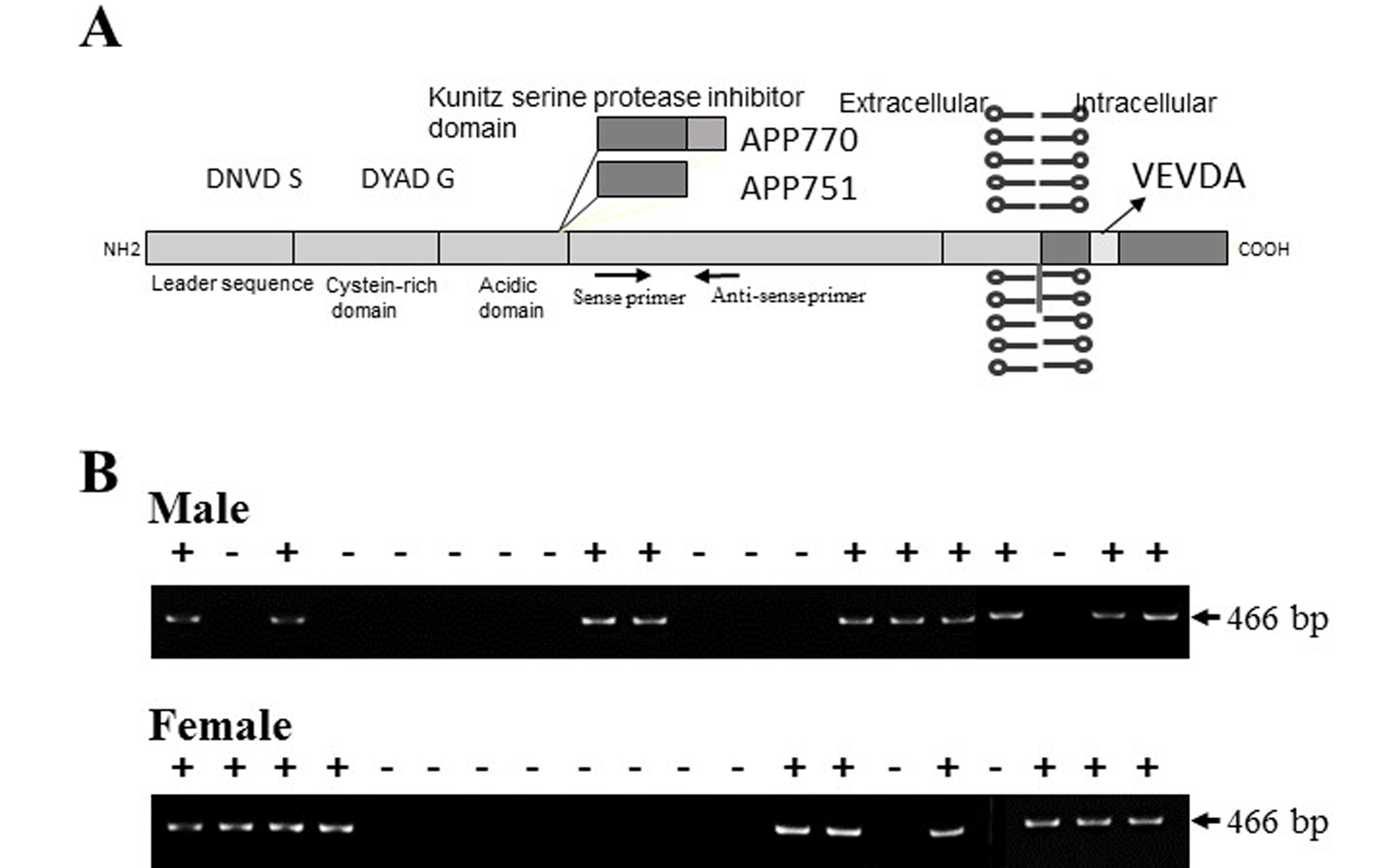

Supplement: S1 Fig — (A) Tg2576 mice have the mutant human APP gene 695 amino acid isoform and with a double mutation (Lys670→Asn and Met671→Leu). (B) DNA-PCR analysis were performed on genomic DNA isolated from the tail of founder mouse, and the 442 bp of products were shown in Tg mice carrying the APPsw transgenes. (TIF) [file pone.0215205.s001.tif]
