# Supplementary material for: Constipation in Tg2576 mice model for Alzheimer’s disease associated with dysregulation of mechanism involving the mAChR signaling pathway and ER stress response
Source: PLoS One. 2019 Apr 12;14(4):e0215205. doi: 10.1371/journal.pone.0215205 (PMC6461235; doi:10.1371/journal.pone.0215205)
Supplement: S1 Table — (DOCX) [file pone.0215205.s002.docx]

|  | | | 1 | 2 | 3 | 4 | 5 | 6 | 7 | 8 | 9 | 10 | Average | Stdev |
| --- | --- | --- | --- | --- | --- | --- | --- | --- | --- | --- | --- | --- | --- | --- |
| Stool number | Male | Non | 49 | 30 | 40 | 66 | 52 | 36 | 32 | 43 | 40 | 60 | 44.80 | 11.22 |
|  |  | Tg | 9 | 21 | 30 | 8 | 27 | 22 | 21 | 28 | 27 | 9 | 20.20 | 8.08 |
|  | Female | Non | 34 | 34 | 49 | 34 | 25 | 42 | 33 | 37 | 24 | 56 | 36.80 | 9.41 |
|  |  | Tg | 14 | 13 | 15 | 29 | 26 | 27 | 12 | 9 | 11 | 15 | 17.10 | 6.95 |
| Stool weight | Male | Non | 0.33 | 0.43 | 0.38 | 0.87 | 0.48 | 0.69 | 0.42 | 0.36 | 0.46 | 0.51 | 0.49 | 0.16 |
|  |  | Tg | 0.11 | 0.16 | 0.38 | 0.27 | 0.40 | 0.10 | 0.37 | 0.12 | 0.28 | 0.12 | 0.23 | 0.12 |
|  | Female | Non | 0.35 | 0.30 | 0.35 | 0.28 | 0.34 | 0.31 | 0.30 | 0.51 | 0.43 | 0.35 | 0.36 | 0.06 |
|  |  | Tg | 0.27 | 0.22 | 0.27 | 0.19 | 0.22 | 0.21 | 0.27 | 0.21 | 0.22 | 0.29 | 0.24 | 0.03 |

**S1 Table. Number of weight of stools of Tg2576 mice**
