# Supplementary material for: Expression of Concern: Constipation in Tg2576 mice model for Alzheimer’s disease associated with dysregulation of mechanism involving the mAChR signaling pathway and ER stress response
Source: PLoS One. 2026 Aug 26;21(8):e0356955. doi: 10.1371/journal.pone.0356955 (PMC13514039; doi:10.1371/journal.pone.0356955)

Actin

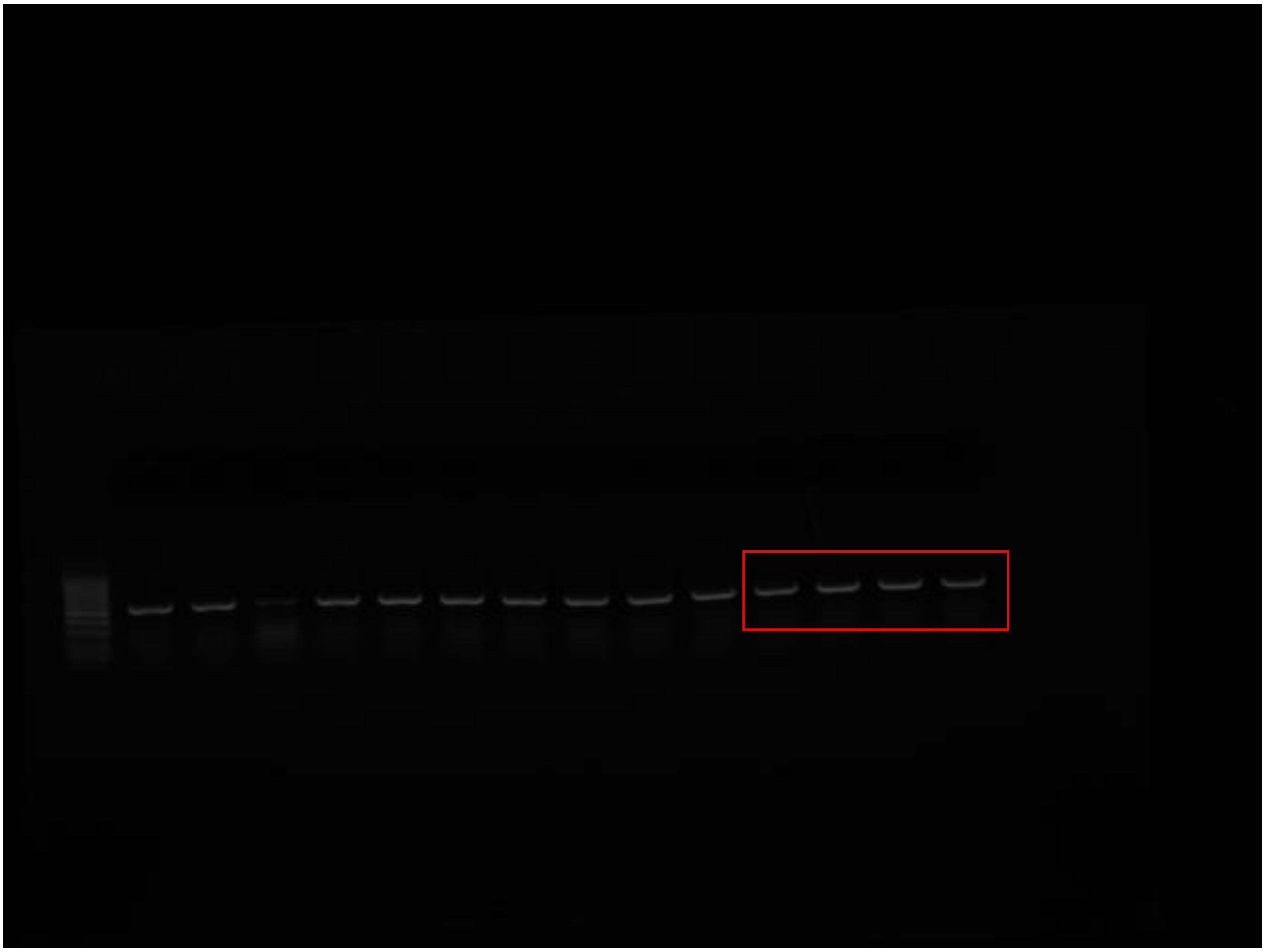

M1

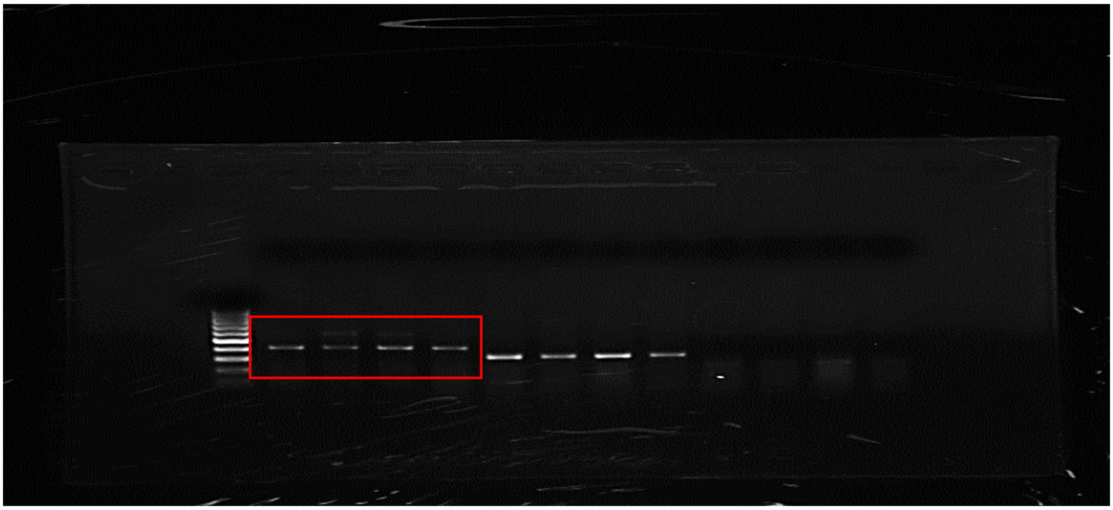

M2

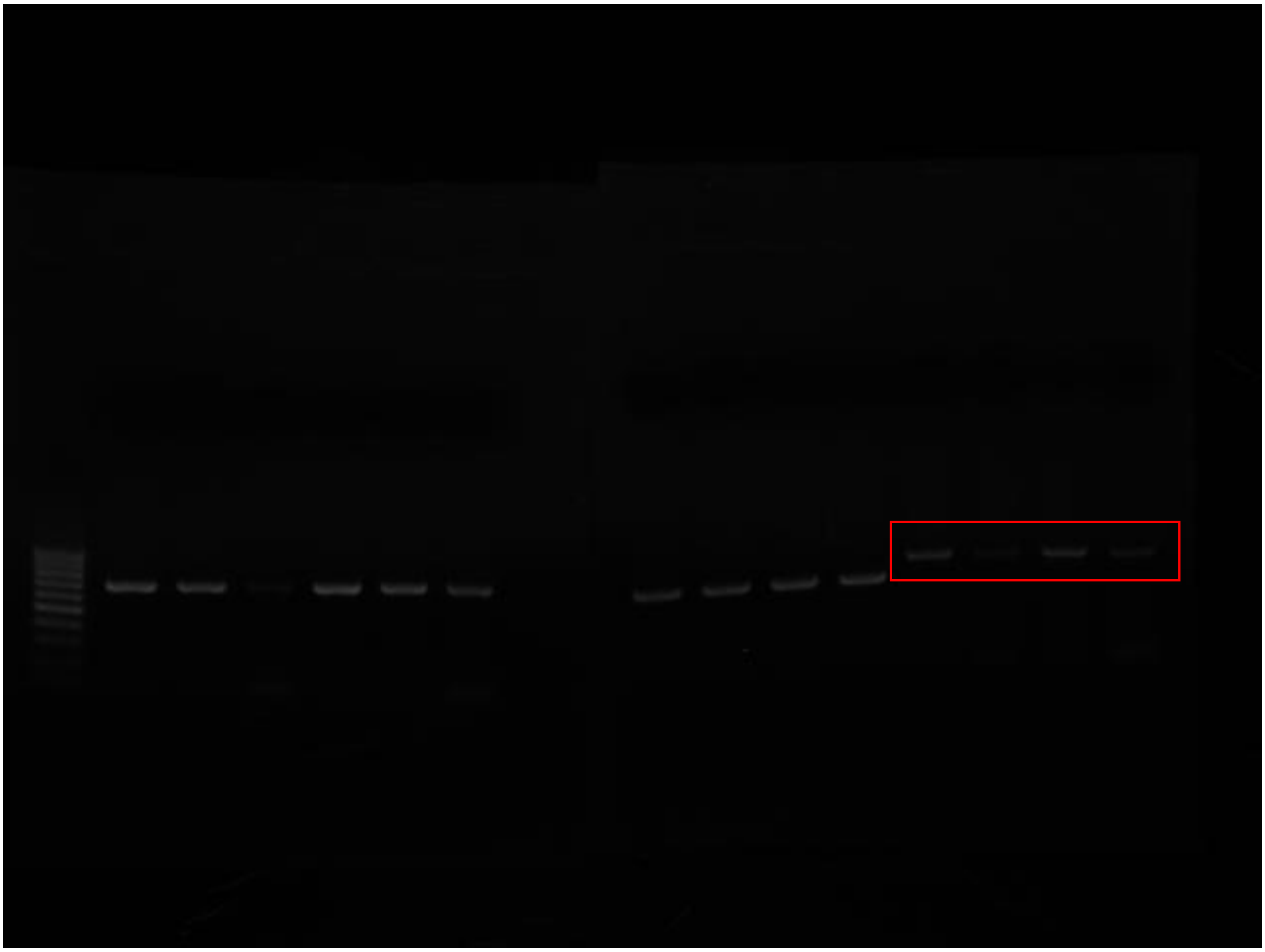

M3

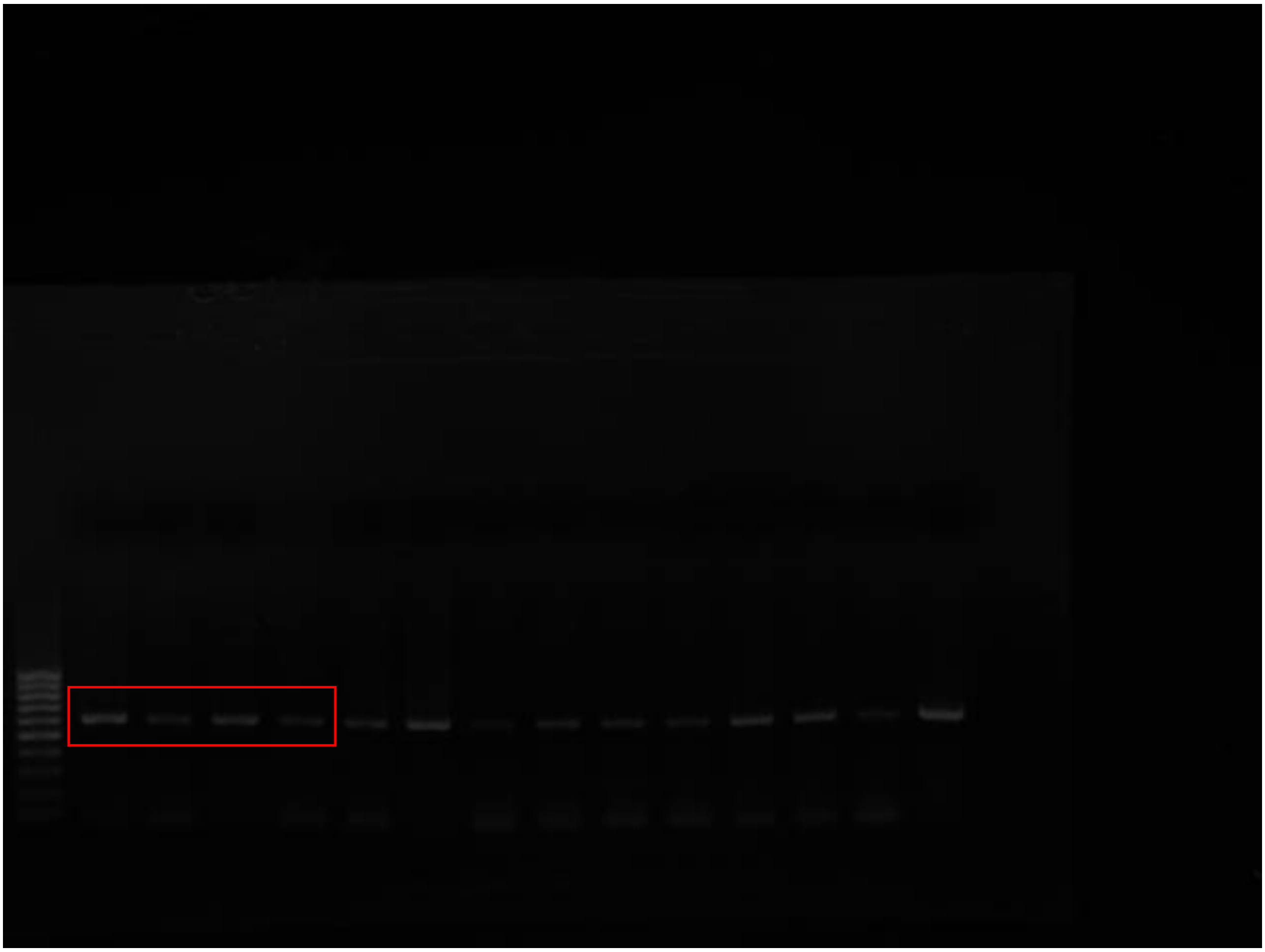

M4

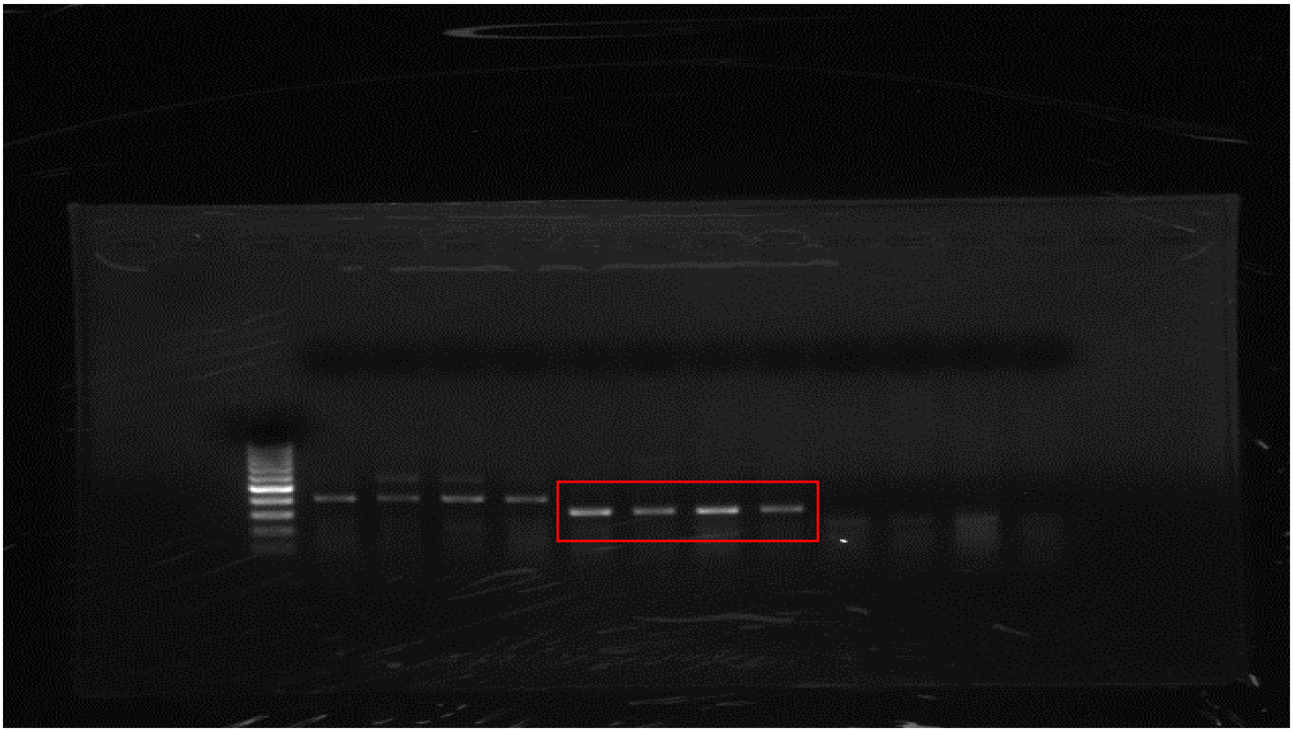

Supplement: Supplementary file 1 [file pone.0356955.s002.zip › S1 File/Fig 6/Figure 6A.pdf]
